# Immune response to COVID-19 vaccination in a population with a history of elevated exposure to per- and polyfluoroalkyl substances (PFAS) through drinking water

**DOI:** 10.1038/s41370-023-00564-8

**Published:** 2023-06-19

**Authors:** Jordan M. Bailey, Ling Wang, Jennifer M. McDonald, Jennifer S. Gray, Joshua G. Petrie, Emily T. Martin, David A. Savitz, Timothy A. Karrer, Keri A. Fisher, Matthew J. Geiger, Elizabeth A. Wasilevich

**Affiliations:** 1 Division of Environmental Health, Michigan Department of Health and Human Services, Lansing, MI USA; 2 Department of Medicine, College of Human Medicine, Michigan State University, East Lansing, MI USA; 3 Center for Clinical Epidemiology and Population Health, Marshfield Clinic Research Institute, Marshfield, WI USA; 4 Department of Epidemiology, University of Michigan School of Public Health, Ann Arbor, MI USA; 5 Department of Epidemiology, Brown University School of Public Health, Providence, RI USA; 6 Division of Chemistry and Toxicology, Michigan Department of Health and Human Services, Lansing, MI USA

**Keywords:** PFAS, Epidemiology, Health studies, Emerging contaminants

## Abstract

**Background:**

Exposure to per- and polyfluoroalkyl substances (PFAS) has been linked to lower vaccine-induced antibody concentrations in children, while data from adults remains limited and equivocal. Characteristics of PFAS exposure and age at vaccination may modify such effects.

**Objective:**

We used the mass administration of novel COVID-19 vaccines to test the hypothesis that prior exposure to environmentally-relevant concentrations of PFAS affect antibody response to vaccines in adolescents and adults.

**Methods:**

Between April and June 2021, 226 participants aged 12–90 years with a history of exposure to PFAS in drinking water and who received an mRNA COVID-19 vaccine participated in our prospective cohort study. SARS-CoV-2 anti-spike and anti-nucleocapsid antibodies (IgG) were quantified before the first and second vaccine doses and again at two follow-ups in the following months (up to 103 days post dose 1). Serum PFAS concentrations (n = 39 individual PFAS) were measured once for each participant during baseline, before their first vaccination. The association between PFAS exposure and immune response to vaccination was investigated using linear regression and generalized estimating equation (GEE) models with adjustment for covariates that affect antibody response. PFAS mixture effects were assessed using weighted quantile sum and Bayesian kernel machine regression methods.

**Results:**

The geometric mean (standard deviation) of perfluorooctane sulfonate and perfluorooctanoic acid serum concentrations in this population was 10.49 (3.22) and 3.90 (4.90) µg/L, respectively. PFAS concentrations were not associated with peak anti-spike antibody response, the initial increase in anti-spike antibody response following vaccination, or the waning over time of the anti-spike antibody response. Neither individual PFAS concentrations nor their evaluation as a mixture was associated with antibody response to mRNA vaccination against COVID-19.

**Impact statement:**

Given the importance of understanding vaccine response among populations exposed to environmental contaminants and the current gaps in understanding this relationship outside of early life/childhood vaccinations, our manuscript contributes meaningful data from an adolescent and adult population receiving a novel vaccination.

## Introduction

Exposure to PFAS, a large group of highly-fluorinated and environmentally-persistent compounds, is relevant to much of the global population [1–6]. Subpopulations of highly-exposed workers and residents living near sources of PFAS contamination have also been described [7, 8]. The impact of exposure to per- and polyfluoroalkyl substances (PFAS) on the immune system is of growing concern. Associations between exposure to some PFAS and decreased antibody response to vaccines have been used by the European Food Safety Authority [9] and the U.S. Environmental Protection Agency [10] in the development of toxicity values used for PFAS risk assessment. Moreover, the 2022 U.S. National Academies of Sciences, Engineering, and Medicine report [11] similarly concluded that there is likely sufficient evidence that PFAS exposure is associated with decreased antibody response to vaccines. The report notes, however, that there is currently insufficient evidence of an increase in risk or severity of infection or differences in vaccine effectiveness among those exposed to PFAS.

Environmental exposure to particular PFAS, notably perfluorooctanesulfonic acid (PFOS) and perfluorooctanoic acid (PFOA), has repeatedly been linked with adverse health outcomes. This observation has been made in various contexts, including studies of the general population and highly exposed communities, which have involved both adults and children. From these, changes in immune [12–14], cardiovascular [15], kidney [16, 17], liver [18, 19] and thyroid function [20] have been described, among other health effects. However, the impacts of exposure to mixtures of PFAS, the timing of exposure, and individual variation in susceptibility are not well understood for most health outcomes. The current understanding of how PFAS impacts immune function among adults, in particular, remains limited and equivocal.

Although serum PFAS concentrations have been associated with reduced antibody response following routine immunizations (e.g., diphtheria [21–24] and tetanus [22, 23]) among children, studies among adult populations receiving vaccinations have yielded less consistent findings, which may depend upon the type of vaccination studied [23, 25–27]. To our knowledge, only one study to-date has examined the relationship between individual serum PFAS concentration and the humoral immune response to vaccinations against SARS-CoV-2 specifically, and did so in an occupationally exposed population, showing small inverse trends [28]. Governmental agencies and the U.S. National Academies of Sciences, Engineering and Medicine have stated the literature from adult populations is moderate or sufficient to conclude that PFAS alters antibody response to vaccination, in general [11].

The recent and ongoing mass vaccination against SARS-CoV-2, which is responsible for coronavirus disease 2019 (COVID-19), has presented a unique opportunity to study the impact of PFAS serum concentration on antibody response to vaccine administration among adolescents and adults. Identifying a reduced response would raise concerns with the level of protection against COVID-19 in populations with a history of elevated PFAS exposure. The objective of the present study, therefore, was to evaluate the immunogenicity of COVID-19 vaccination through repeated measurements of anti-spike (anti-S) antibodies (IgG) in a cohort of adolescents and adults with a known history of exposure to PFAS-contaminated drinking water for whom we measured total PFAS exposure (from all sources) via serum.

## Methods

### Study population

In April–June 2021, we recruited individuals from an existing cohort, the Michigan PFAS Exposure and Health Study (MiPEHS), to participate in the PFAS and Antibody Response to COVID-19 Vaccine Study. MiPEHS is a longitudinal study examining health outcomes and serum PFAS concentrations in a cohort from two communities in western Michigan impacted by PFAS-contaminated drinking water. To be eligible for MiPEHS, and by extension, the COVID-19 antibody response study, participants were required to have lived in either of two specific communities (referred to below as “geographical sites”) between 2005 and 2018, which corresponds to when contamination is thought to have impacted drinking water (while still being detectable in serum samples). Contamination is thought to have resulted from nearby landfills and could have begun decades before discovery. Elevated PFAS concentrations were demonstrated at the tap and/or at the municipal source for most participants in 2018, at which time exposure mitigation and remediation efforts were undertaken to reduce or remove PFAS from drinking water. Potential participants were contacted directly by phone and mail for recruitment. A study website (www.Michigan.gov/DEHBio) was used to promote the study.

Participants receiving either mRNA COVID-19 vaccine (Moderna or Pfizer-BioNTech) available under emergency use authorization or approved by the Food and Drug Administration (FDA) were included in analyses. COVID-19 vaccines were not provided as part of participation in this study. We excluded pregnant females; those who were recently (i.e., within <90 days) treated for COVID-19 infection with convalescent plasma, intravenous immunoglobulin, or monoclonal antibody; were actively receiving immunosuppressive therapies for cancer, organ transplant, or autoimmune disease; or were undergoing dialysis.

**Fig. 1:**
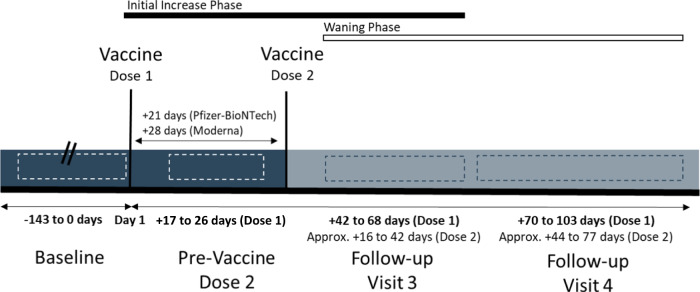
Schedule of blood sampling relative to vaccine administration. Participants were asked to provide a blood sample before their first and second vaccine doses as well as blood samples during two follow-up visits, anchored around 30 and 60 days, respectively, after their second dose of the 2-dose vaccine series. Target windows depicted in this diagram are inclusive of both the different schedules for Pfizer-BioNTech (+21 day) and Moderna (+28) second doses as well as a 7-day buffer granted to participants for scheduling convenience, participants arriving outside of this schedule were included if their blood draws fell within the wider windows for follow-up depicted here. Baseline data are referred to as “day 0” throughout, including when the data are presented graphically.

Each participant attended up to four study office appointments for data and blood sample collection, depending on when they enrolled in the study relative to their vaccination dates. These office visits were planned to include a baseline (pre-vaccination) visit, a pre-second vaccination dose visit, a visit approximately 1–2 months after the second vaccine dose, and a visit approximately 2–3 months after the second vaccine dose (Fig. 1). Participants could join the study at any point within this schedule of visits. If they joined after their first or second vaccination dose we used their pre-vaccination MiPEHS sample as their baseline measurements for this study. The windows for the follow-up visits reflect the different vaccine schedules for Pfizer-BioNTech’s (21 days between first and second dose) and Moderna’s (28 days between first and second dose) vaccine as well as participant availability and willingness to attend follow-up appointments. Participants received a scaled incentive per study office appointment up to $135 total for all study participation.

All study participants provided written informed consent to participate. All aspects of the study were approved by and conducted in compliance with the Michigan Department of Health and Human Services Institutional Review Board.

### Data collection

During study office visits, we obtained consent from participants (parents/guardians of minors), administered a survey, collected blood samples, and measured height and weight. The survey administered at the first visit collected information on demographics, prior diagnosis of health conditions that could affect antibody response (e.g., diabetes), their history of positive COVID-19 test(s), current COVID-19 symptoms, whether they came into close contact with someone diagnosed with COVID-19, and their planned or completed date of COVID-19 vaccine and their vaccine type (or brand). Surveys administered at all subsequent visits queried COVID-19 vaccine history and vaccine type(s) administered, updated information for diagnoses that could affect antibody response, updated other vaccine information, updated information regarding positive COVID-19 test(s), current COVID-19 symptoms, and whether they came into close contact with someone diagnosed with COVID-19 since their last study office visit. Self-reported vaccination dates and vaccine types were confirmed using information in the state’s immunization registry, the Michigan Care Improvement Registry. When there was disagreement between self-report and the immunization registry data, the immunization registry data were used (except in fewer than 5% of cases where we failed to match immunization registry data to study participants and self-report was used). Participants also provided consent to access other records and specimens related to their MiPEHS participation, which included blood samples.

During study office visits for this study, trained phlebotomists collected non-fasting blood samples from participants. On a participant’s first study office visit, two tubes of blood (BD Vacutainer Serum Tubes) were collected, one for PFAS testing and one for antibody quantification. On participant’s follow-up visits, only one tube of blood was collected, which was for antibody quantification. This did not change depending on when participants joined the study. As noted previously, if participants’ first appointment was after their vaccination series began, their pre-vaccination MiPEHS sample served as their baseline measure for antibody quantification. Their PFAS measurement was always collected at their first visit for the present study.

### Serum PFAS testing

PFAS concentrations in serum samples were measured by the MDHHS Bureau of Laboratories using reverse-phase high performance liquid chromatography (RP-HPLC) tandem mass spectrometry (MS/MS). Sample preparation prior to analysis entailed isotope dilution and the addition of acetonitrile to precipitate proteins. Samples were further cleaned up using a 96-well filtration plate and concentrated 20-fold prior to analysis. Sample preparation and analytical measurements were conducted using a validated method and followed strict quality control and quality assurances in accordance with College of American Pathologist (CAP) and Clinical Laboratory Improvement Amendments (CLIA) regulations. We measured 39 PFAS, plus branched isomers of PFOS, PFOA and PFHxS. For these, the sum of branched and linear isomers was calculated to create a “total” concentration, which was used in all analyses. Native and isotopically labelled standards were purchased from Wellington Laboratories Inc, Guelph Ontario Canada. Analysis was performed using Shimadzu LC-MS 8060 mass spectrometers. The full technical details of this method will be published separately. Supplementary Table S1 includes the full list of PFAS measured with their corresponding limit of quantification (LOQ). For the analyses, values below the LOQ were imputed by the LOQ divided by the square root of 2 [29, 30].

### Serum SARS-CoV-2 antibody testing

Using protocols developed by the National Institute of Allergy and Infectious Diseases Vaccine Research Center [31, 32], serum specimens were tested by enzyme-linked immunosorbent assays (ELISA) to measure antibody binding to the SARS-CoV-2 spike and nucleocapsid proteins, which is expressed as area under the curve (AUC). Anti-nucleocapsid IgG (anti-N) and anti-spike IgG (anti-S) were measured in pre-vaccination samples to evaluate for previous infection with SARS-CoV-2. Anti-S was used as the primary measure of immunogenicity following vaccination; an anti-N response would not be expected following vaccination.

Briefly, 96-well flat bottom plates were coated with SARS-CoV-2 spike or N proteins overnight. Plates were then blocked for one hour with 3% milk solution in PBS-tween. The blocking solution was then washed off and the test serum samples were added in duplicate at 1:100 dilution. Eight-point serial fourfold dilutions were then performed on the test samples before incubating for two hours. A pooled positive control using the same dilution scheme as the test samples and 8 blank wells were also included on each plate. After incubation, plates were washed, and the detection antibody was added and incubated for one hour. Plates were then washed before adding TMB substrate for 10 min before stopping. Optical density (OD) readings were then made with an absorbance reader. Spike protein antigen was produced by the Center for Structural Biology in the Life Sciences Institute at the University of Michigan. N protein antigen was purchased from Fisher Scientific (Invitrogen RP-87665). All ELISA assays were performed at the University of Michigan School of Public Health. AUC endpoints were calculated from OD readings using Prism software (GraphPad Prism, San Diego, CA).

Thresholds for anti-N and anti-S seropositivity were determined from a set of existing serum specimens collected from 10 individuals enrolled in a pre-pandemic study of influenza vaccine effectiveness and 98 convalescent serum specimens collected from individuals with confirmed SARS-CoV-2 infection. For anti-N, an AUC threshold of 1522 had a sensitivity of 78% with 100% specificity in this dataset. For anti-S, an AUC threshold of 252 had a sensitivity of 98% with 100% specificity in this dataset.

### Statistical analyses

Our analyses were limited to individuals who came to at least one study office visit where they provided a blood sample, and who received both doses of an mRNA COVID-19 vaccine on schedule (as confirmed by immunization registry records). PFAS serum measurements corresponded to samples collected during the participants first study office visit. We log_2_ transformed all PFAS variables because PFAS concentrations were right-skewed. PFAS detected in at least 60% of participants were used in subsequent analyses. We assessed correlations among serum PFAS analytes using Spearman correlation; coefficients with values of 0.70 or higher were considered highly correlated (see Supplementary Fig. S1).

Anti-S and anti-N antibody measurements were obtained from samples collected during each visit a participant attended. We also log_2_ transformed antibody variables to normalize the distributions. Participants were categorized as having a history of COVID-19 infection (i.e., “recovered”) if they reported a history of COVID-19 infection, had an anti-N antibody AUC above the threshold of 1522 at any point, or had an anti-S antibody AUC above the threshold of 252 in their pre-vaccine (i.e., baseline) sample. Prior infection status was included in statistical models to account for any influence of prior infection on the relationship between PFAS serum concentrations and antibody response to vaccination. Participants who did not meet any of these criteria were considered naive to COVID-19 infection.

We assumed that there would be initial exponential increases following the two doses of mRNA vaccine [33] followed by a waning phase in anti-S antibody AUC, so we performed separate analyses to capture the initial phase and second phase of the anti-S antibody response separately. The initial increase phase includes baseline data through visit 3 (where visit 3 corresponds to day 42 through 68 days after first vaccine dose; which is 16–42 days after second vaccine dose) and the waning second phase includes data from visit 3 through visit 4 (where visit 4 corresponds to day 70 through 103 days after first vaccine dose; which is 44–77 days after second vaccine dose). Baseline data are referred to as “day 0” throughout, including when the data are presented graphically.

First, we examined peak antibody response following vaccination, at visits 3 and 4, using linear regression. We then carried out cross-sectional analyses using the change over time (delta) in log_2_ transformed anti-S antibody AUCs as outcomes during both phases (initial increase and later waning), such that we considered: (1) change in log_2_ transformed anti-S antibody AUC between baseline and the third blood draw (42–68 days after first vaccine dose) (∇_1_ = log2 *Spike AUC at visit*3 − log2 *Spike AUC at baseline*) and (2) change in log_2_ transformed anti-S antibody AUCs between the third blood draw (42 to 68 days after first vaccine dose) and the fourth blood draw (70–103 days after first vaccine dose) (∇_2_ = log2 *Spike AUC at visit*4 − log2 *Spike AUC at visit*3). For these cross-sectional analyses, three modeling methods are employed: (1) linear regression by including each serum PFAS as one independent variable; (2) Weighted Quantile Sum (WQS) regression by creating a single score (the weighted quantile sum) that summarizes the overall exposure of all PFAS measured, and including this score in the regression model to evaluate the overall effect of the PFAS mixture on the change in log_2_ anti-S antibody AUCs; and (3) Bayesian Kernel Machine Regression (BKMR) which flexibly models the nonlinear relationship between each PFAS and differences in log_2_ transformed anti-S antibody AUCs. All cross-sectional analyses controlled for sex, age, race, past COVID-19 infection status, vaccine type and geographical site of the participants as covariates.

To further understand the change in antibody response over time, we then used a generalized estimating equations (GEE) model to estimate the association between serum PFAS concentration (with each serum PFAS included as one independent variable) and changes in anti-S antibody AUC as repeated measures over time within each phase of the immune response. The GEE model accounts for within-subject correlation between baseline and follow-up measures of anti-S antibody levels. Specifically, the GEE estimation used in this model is:

$$y_{it} = \, 	 \alpha _i + \beta _1 \times PFAS_i + \beta _2 \times I_{i,{{{{{\rm{Visit3}}}}}}} + \beta _3 \times \left( {I_{i,{{{{{\rm{Visit3}}}}}}} \times PFAS_i} \right) + \gamma \\ 	 \times \,{\it{{{{{\rm{Other}}}}}}}\,{\it{{{{{\rm{covariates}}}}}}}_i + \varepsilon _{it}\,{{{{{{{\mathrm{for}}}}}}}}\,{{{{{{{\mathrm{the}}}}}}}}\,{{{{{{{\mathrm{initial}}}}}}}}\,{{{{{{{\mathrm{increase}}}}}}}}\,{{{{{{{\mathrm{phase}}}}}}}},$$

and

$$y_{it} =\, 	 \alpha _i + \beta _1 \times PFAS_i + \beta _2 \times {\it{{{{{\rm{days}}}}}}}\,{\it{{{{{\rm{from}}}}}}}\,{\it{{{{{{\rm{first}}}}}}}}\,{\it{{{{{{\rm{vaccine}}}}}}}}_i + \beta _3\\ 	 \times \,\left( {{\it{{{{{{\rm{days}}}}}}}}\,{\it{{{{{{\rm{from}}}}}}}}\,{\it{{{{{{\rm{first}}}}}}}}\,{\it{{{{{{\rm{vaccine}}}}}}}}_i \times PFAS_i} \right) + \gamma \times {\it{{{{{{\rm{Other}}}}}}}}\,{\it{{{{{{\rm{covariates}}}}}}}}_i\\ 	 + \,\varepsilon _{it}{{{{{{{\mathrm{for}}}}}}}}\,{{{{{{{\mathrm{the}}}}}}}}\,{{{{{{{\mathrm{second}}}}}}}}\,{{{{{{{\mathrm{waning}}}}}}}}\,{{{{{{{\mathrm{phase}}}}}}}},$$

where *y*_*it*_ are the log_2_ anti-S antibody AUC for individual *i*, at timepoint, *t*. Since during the initial increase phase, the change of anti-S antibody AUC is not expected to have a linear relationship over time, we estimated the overall anti-S antibody AUC change from baseline to visit 3 (so the unit of *t* is visit). However, during the second (waning) phase, the waning of anti-S antibody AUC is expected to have a linear change by days, so we estimated the daily change of anti-S antibody AUC instead of overall change during this phase. The coefficient before the interaction term, *β*_3_, captured the effect of PFAS on anti-S antibody AUC changing over time. Sex, age, race, past COVID-19 infection status, vaccine type and geographic site of the participants were included in the GEE model to control for possible confounding effects.

All continuous covariables were summarized as mean ± standard deviation and categorical variables were summarized as absolute frequency and percentage. A *P* value of <0.05 was considered statistically significant in all analyses. All analyses were performed using R version 4.1.1. and R packages “gee,” “gWQS” and “bkmr” were used for GEE, WQS and BKMR models, respectively.

## Results

### Descriptive results

We enrolled a total of 251 participants; of these, 243 received an mRNA COVID-19 vaccine (Pfizer-BioNTech or Moderna), the remaining 17 received a non-mRNA vaccine (Johnson & Johnson) and were excluded from further analyses. A further 8 participants received their second dose of their mRNA vaccine outside of the target window recommended by the CDC or they had no record of a second dose and were excluded from further analyses due to an incomplete or potentially invalid vaccination history. A total of 226 participants provided blood samples and had complete/valid mRNA vaccination histories and were included in the analyses (see Supplementary Fig. 2). Participants ranged in age from 12 to 90 years. Age was not found to interact with PFAS and antibody response (p < 0.05). All participants resided in the two selected geographical sites, which were recently impacted by PFAS-contaminated drinking water. Table 1 shows demographic characteristics of participants by PFOS and PFOA serum concentration and Table 2 describes serum anti-S antibody AUC by visit for key participant demographics.

**Table 1:** Demographic characteristics of the study population who received a valid 2-dose series of mRNA vaccine and provided a serum sample for antibody quantification (*N* = 226).

	*n* (%)	PFOA µg/L GM (GSD)	PFOS µg/L GM (GSD)
Overall	226 (100%)	3.90 (4.90)	10.49 (3.22)
Age, years			
12–20	15 (6.6%)	3.29 (3.78)	9.21 (3.35)
21–40	29 (12.8%)	5.53 (4.53)	10.18 (2.53)
41–60	71 (31.4%)	3.42 (5.42)	8.58 (3.6)
>60	111 (49.1%)	3.97 (4.85)	12.18 (3.1)
Sex			
Male	95 (42.0%)	4.71 (4.76)	12.43 (3.16)
Female	131 (58.0%)	3.39 (4.95)	9.21 (3.22)
Race			
White	211 (93.3%)	4.01 (4.95)	10.59 (3.19)
Other	15 (6.7%)	2.72 (4.14)	8.67 (3.46)
Site			
1	108 (47.8%)	10.28 (4.18)*	16.78 (3.16)*
2	118 (52.2%)	1.62 (3.16)	6.75 (2.75)

An asterisk (*) is used to denote *p* values less than 0.05 for comparisons within each group. Fewer than 7 participants reported their race to be any race or combination of races other than white and are reported as “other”, for example, Black, Indigenous, or other person of color. Fewer than 7 participants reported an ethnicity other than non-Hispanic, therefore, ethnicity is not reported here.

*µg/L* microgram per liter, *GM* geometric mean, *GSD* geometric standard deviation.

**Table 2:** Anti-S antibody AUC of the study population who received a valid 2-dose series of mRNA vaccine and provided a serum sample for antibody quantification (*n* = 224).

	Baseline (pre-vaccine)		Visit 2 (16–26 days)		Visit 3 (42–68 days)		Visit 4 (70–103 days)	
	*n*	Log (2) Anti-S AUC Mean (SD)	*n*	Log (2) Anti-S AUC Mean (SD)	*n*	Log (2) Anti-S AUC Mean (SD)	*n*	Log (2) Anti-S AUC Mean (SD)
Overall	130	5.99 (5.73)	37	13.31 (3.12)	140	15.64 (2.70)	208	14.82 (2.45)
Age, years								
12–20	9	7.38 (3.06)	7	13.9 (1.93)	9	17.66 (1.57)*	13	17.35 (1.49)*
21–40	20	3.07 (7.26)	5	14.24 (4.38)	22	16.21 (1.8)	26	15.83 (1.73)
41–60	42	6.03 (5.03)	13	12.11 (3.51)	53	15.59 (2.52)	64	14.92 (2.01)
>60	59	6.74 (5.72)	12	13.89 (2.62)	56	15.15 (3.14)	105	14.19 (2.66)
Sex								
Male	57	6.09 (6.14)	19	14.27 (2.84)	64	15.85 (2.98)	86	14.7 (2.57)
Female	73	5.91 (5.42)	18	12.31 (3.17)	76	15.47 (2.46)	122	14.9 (2.38)
Race								
White	122	6.17 (5.64)	34	11.77 (2.56)	132	14.55 (1.2)	198	13.99 (1.89)
Other	8	3.16 (6.75)	3	13.45 (3.16)	8	15.71 (2.76)	10	14.86 (2.47)
Prior COVID-19 Infection								
Naive	66	2.21 (4.8)*	28	12.57 (2.9)*	85	15.34 (2.9)	124	14.78 (2.52)
Recovered	64	9.89 (3.6)	9	15.61 (2.77)	55	16.11 (2.77)	84	14.88 (2.36)
Vaccine type								
Pfizer-BioNTech	105	5.96 (5.75)	30	12.96 (3.21)	117	15.72 (2.78)	166	14.79 (2.33)
Moderna	25	6.09 (5.76)	7	14.82 (2.35)	23	15.26 (2.28)	42	14.91 (2.9)
Site								
1	108	5.9 (5.51)	17	14.46 (3.01)*	59	16.14 (2.03)	100	15 (2.38)
2	118	6.07 (5.98)	20	12.34 (2.95)	81	15.28 (3.06)	108	14.64 (2.52)

An asterisk (*) is used to denote *p* values less than 0.05 for comparisons within each group. Fewer than 7 participants reported their race to be any race or combination of races other than white (e.g., Black) and are reported as “other”. Fewer than 7 participants reported an ethnicity other than non-Hispanic, therefore, ethnicity is not reported here. “Days” describe the number of days after administration of the first vaccine dose.

*AUC* area under the curve, *SD* standard deviation.

PFOS and PFOA were detected in 100% of participants and therefore are described in detail. PFHxS, MeFOSAA, PFDA, PFHpA, PFHpS, PFNA, PFPeS, PFUnA, and PFECHS were detected in 60% or more of the participants; all 11 of these PFAS are described in detail in the supplementary materials (e.g., Supplementary Table S2) and are included in the mixture analyses described below. Supplementary Fig. S1 shows the Pearson correlations among the 11 PFAS detected in at least 60% of participants. Both serum PFOS and PFOA concentrations were higher among males than females (Table 1).

Among all PFAS detected, the highest mean serum concentration was for PFOS, at 10.49 µg/L, followed by PFOA at 3.9 µg/L (Table 1). Overall, participants had substantially higher blood concentrations of PFOS and PFOA compared to national exposure data (e.g., 95th percentile of PFOS among study participants was 79.92 µg/L, while NHANES (2017–2018) reports a 95th percentile of 14.6 µg/L with a geometric mean of 4.25 µg/L; 95th percentile of PFOA among study participants was 61.81 µg/L, while NHANES reports a 95^th^ percentile of 3.8 µg/L and a geometric mean of 1.42 µg/L). Note, our PFAS method and that used by CDC for NHANES is similar (both use isotope dilution) and our laboratory has a lower reporting limit (0.025 ng/ml) compared to the CDC laboratory (0.10 ng/ml), permitting reasonable confidence that comparisons between our results and those of NHANES are appropriate.

**Fig. 2:**
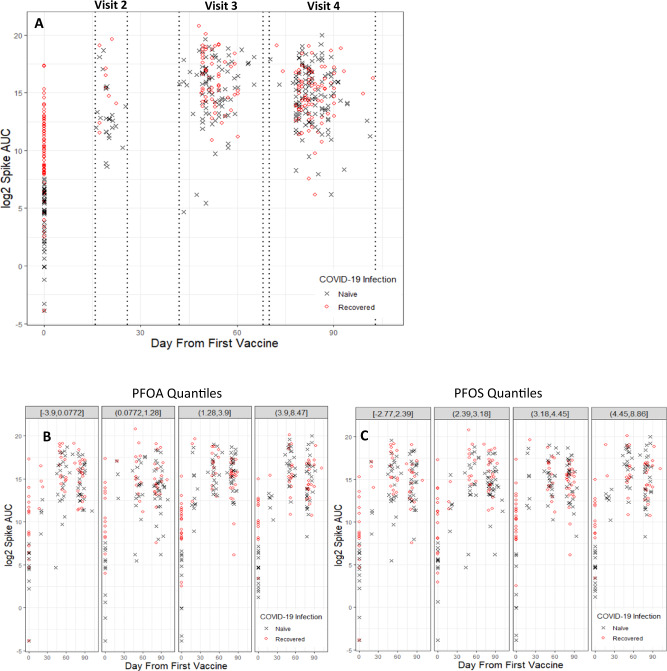
Log_2_ anti-S antibody AUC as a function of days since first vaccination dose (Pfizer-BioNTech or Moderna) for participants recovered from a prior COVID-19 infection or naive to COVID-19. **A** All data collected as a function of time (days from first vaccine are on the x-axis) for both the participants naive to (blue) and recovered from (red) prior COVID-19 infection. The longitudinal data by log_2_ PFOA (**B**) and PFOS (**C**) quantile, boundaries of each quantile are displayed in gray shaded header above each panel.

At baseline, 66 out of 130 (50.8%) participants had no prior COVID-19 infection (Table 2). Figure 2 (panel A) shows the longitudinal log_2_ anti-S antibody AUCs over the course of our data collection period, for individuals with and without prior COVID-19 infection. Figure 2B, C further displays these longitudinal data by log_2_ PFOA (panel B) and log_2_ PFOS (panel C) quantiles and Supplementary Fig. S4 shows this for all other log_2_ PFAS quantiles.

### Peak post-vaccination anti-S antibody AUC (by PFAS concentration)

We used linear regression to assess peak post-vaccination geometric mean anti-S antibody AUC by PFAS concentration. Supplementary Fig. S3 displays the longitudinal anti-S antibody AUC for visit 3 (panels A and C) and visit 4 (panels B and D) by log_2_ transformed PFOA (panels A and B) and log_2_ transformed PFOS (panels C and D) quantiles. Overall geometric mean (SD) log_2_ anti-S antibody AUC for visit 3 was 15.64 (2.70) and for visit 4 was 14.82 (2.45) (see Table 2). Geometric mean anti-S antibody AUC at visit 3 and visit 4 did not differ by serum PFAS concentration (all *p* values > 0.05). Table 3 shows the linear regression results for anti-S AUC at visit 3 and visit 4 for PFOS and PFOA and Supplementary Table S3 shows these results for all other PFAS.

**Table 3:** Linear regression results for anti-S antibody AUC at visit 3 and visit 4 for both PFOA (top) and PFOS (bottom).

	Anti-S antibody AUC at visit 3		Anti-S antibody AUC at visit 4	
	Percent change (95% CI)	*P* value	Percent change (95% CI)	*P* value
PFOA concentration	14.9 (−8.9, 38.7)	0.22	4.6 (−13, 22.1)	0.61
Age	−2.7 (−4.5, −0.9)	<0.01	−3.1 (−4.4, −1.8)	<0.01
Female vs. male	−20.3 (−56.6, 46.2)	0.46	13.7 (−27.9, 79.2)	0.58
Geographical site: 2 vs. 1	−21.1 (−63.2, 69.2)	0.54	−4.4 (−44.9, 65.7)	0.87
History of COVID-19 (naive vs. recovered)	92.9 (3.5, 259.6)	0.04	21.8 (−22.8, 92.1)	0.4
Vaccine Brand (Moderna vs. Pfizer-BioNTech)	−22.5 (−67.3, 83.4)	0.56	−26.3 (−58.1, 29.5)	0.29
PFOS concentration	5.8 (−21.6, 33.3)	0.68	8.5 (−12.4, 29.4)	0.43
Age	−2.7 (−4.5, −0.9)	<0.01	−3.2 (−4.4, −1.9)	<0.01
Female vs. Male	−22.4 (−57. 9, 43.3)	0.42	15 (−27.1, 81.3)	0.55
Geographical site: 2 vs. 1	−37.1 (−67.9, 23.1)	0.18	−5.4 (−41.5, 52.9)	0.82
History of COVID-19 (naive vs. recovered)	94.1 (3.7, 263.3)	0.04	22.1 (−22.6, 92.6)	0.39
Vaccine Brand (Moderna vs. Pfizer-BioNTech)	−16.5 (−64.8, 98)	0.68	−27 (−58.4, 28.3)	0.28

*AUC* area under the curve, *CI* confidence interval, *PFOA* perfluorooctanoic acid (top), *PFOS* perfluorooctanesulfonic acid (bottom).

### Initial increase in anti-S antibody AUC from baseline to visit 3 (by PFAS concentration)

**Fig. 3:**
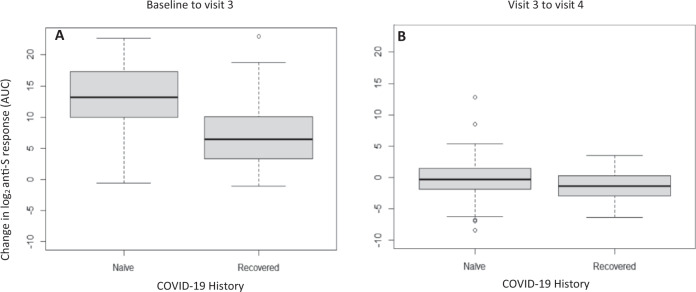
Change in log_2_ anti-S antibody AUC from baseline to the two longest follow-up windows for participants recovered from a prior COVID-19 infection and those naive to COVID-19. Box plots of the change in anti-S antibody AUC between baseline and third visit (**A**) and between the third (42–68 days after first vaccine) and fourth visits (70–103 days after first vaccine) (panel **B**) are shown for participants naive to and recovered from a prior COVID-19 infection. The box indicates interquartile range (IQR = Q–Q1); midline indicates the median. Whiskers are minimum or maximum without outliers. The outliers are the numbers that are below Q1 − 1.5*IQR or above Q3 + 1.5*IQR.

To assess how anti-S antibody AUC changed over time from baseline to visit 3 (or, from day 0 through day 68, which is labed the “first phase” of antibody response), we calculated the change (delta) in log_2_ anti-S antibody AUC from baseline to visit 3 and used this as the outcome in a linear regression model. We found no effect of any serum PFAS concentration (all *p* values > 0.05) (see Table 4) on this measure. A history of prior COVID-19 infection (“recovered”) was associated with a smaller increase in anti-S antibody AUC from baseline to visit 3, for both PFOA (−6.56% change, 95% CI: −8.79, −4.33) and PFOS (−6.61% change, 95% CI: −8.84, −4.39) analyses (see Table 4 and Fig. 3A). Note, however, in Fig. 2A, that the recovered group had higher anti-S antibody AUC at baseline and the recovered and naive groups ultimately reached similar anti-S antibody AUCs.

**Table 4:** Linear regression results for change in anti-S antibody AUC from baseline to visit 3 and the change from visit 3 to visit 4 for both PFOA (top) and PFOS (bottom).

	Log_2_ anti-S antibody AUC change from day 0 to day 68 after first vaccine (initial increase phase)		Log_2_ anti-S antibody AUC change from day 42 to day 103 after first vaccine (waning phase)	
	Percent change (95% CI)	*P* value	Percent change (95% CI)	*P* value
PFOA concentration	−9 (−64.2, 46.1)	0.75	−16.5 (−44.1, 11.2)	0.25
Age	0.5 (−4, 5.4)	0.82	−1 (−3.1, 1.1)	0.35
Female vs. male	−42.5 (−87.2, 158.1)	0.47	63.7 (−19, 230.8)	0.17
Geographical site:2 vs. 1	−59.3 (−93.5, 155.5)	0.34	17.4 (−51.3, 182.8)	0.72
History of COVID-19 (naive vs. recovered)	−98.9 (−99.8, −95)	<0.01	−46.8 (−74.1, 9.1)	0.09
Vaccine Brand (Moderna vs. Pfizer-BioNTech)	111.5 (−73.8, 1605.4)	0.48	10.5 (−60.3, 207.2)	0.85
PFOS concentration	−30.3 (−92.1, 31.5)	0.34	−8 (−39.7, 23.6)	0.62
Age	0.7 (−3.9, 5.4)	0.78	−1(−3.1, 1.1)	0.35
Female vs. Male	−44 (−87.4, 149.5)	0.45	67.3 (−17.7, 240.1)	0.16
Geographical site:2 vs. 1	−63.7 (−92.7, 81.2)	0.22	48.6 (−31.1, 220.5)	0.31
History of COVID-19 (naive vs. recovered)	−99 (−99.8, −95.2)	<0.01	−47.4(−74.4, 8.3)	0.08
Vaccine Brand (Moderna vs. Pfizer-BioNTech)	150.5 (−68.8, 1914)	0.39	3.8(−62.8, 189.6)	0.94

*AUC* area under the curve, *CI* confidence interval, *PFOA* Perfluorooctanoic acid (top), *PFOS* Perfluorooctanesulfonic acid (bottom).

We also employed GEE model to examine the longitudinal change of AUC level from baseline to visit 3 (or, from day 0 through day 68). The GEE models supported the results of linear regression. Log_2_ anti-S antibody AUC increased significantly during this first phase. During this phase, from baseline to visit 3, log_2_ anti-S antibody AUC increased by 9.48 units (95% CI: 8.12, 10.83) in the model with PFOA and by 9.41 units (95% CI: 7.53, 11.29) in the model for PFOS (Table 5). During this first phase, log_2_ anti-S antibody AUC was 4.29 units higher for those with prior infection compared to naive individuals (95% CI: 3.36, 5.23) for the model with PFOA and AUC was 4.31 units higher for those with prior infection compared to naive individuals (95% CI: 3.37, 5.25) for the model with PFOS (see Table 5). No effect of any PFAS was found on this phase of anti-S antibody AUC (all *p* values > 0.05) (see Table 5 and Supplementary Table S4).

**Table 5:** GEE regression results with interaction of PFOA (top) and PFOS (bottom) and days from first vaccine to explain outcome in log_2_ anti-S antibody AUC.

	Day 0 to day 68 after first vaccine^a^ (initial increase phase)		Day 42 to day 103 after first vaccine^b^ (waning phase)	
	Percent change (95% CI)	P-value	Percent change (95% CI)	P-value
Age	−1.2 (−3.1, 0.8)	0.24	−2.7 (−4.1, −1.4)	<0.001
Female vs. Male	−15.2 (−56.1, 63.6)	0.62	2.2 (−31.7, 52.6)	0.92
Geographical site	−20.8 (−63.1, 69.9)	0.55	−10.1 (−42.2, 39.5)	0.64
History of COVID-19 (naive vs. recovered)	1860 (924.2, 3650.7)	<0.01	47.4 (−0.7, 118.9)	0.05
Vaccine Brand (Moderna vs. Pfizer-BioNTech)	−31.7 (−73.1, 73)	0.42	−36.3 (−60.8, 3.5)	0.07
log_2_ serum PFOA concentration	−0.1 (−48.8, 21.9)	0.46	0.4 (−5, 93)	0.08
Visit 3 vs. baseline	71218.1 (27765.8, 182427.5)	<0.01		
Visit 3 × log_2_ serum PFOA concentration	30.4 (−9.7, 70.5)	0.14		
Days from first vaccine			−1.4 (−2.7, 0.7)	0.19
Days from first vaccine × log_2_ serum PFOA concentration			−1 (−1, 0.2)	0.17
Age	−1.2 (−3.2, 0.7)	0.22	−2.7 (−4.1, −1.4)	<0.001
Female vs. Male	−15.2 (−55.9, 63)	0.62	1.4 (−32.2, 51.6)	0.95
Geographical site	−21.6 (−60.6, 55.8)	0.49	−19.9 (−46.8, 21.4)	0.3
History of COVID-19 (naive vs. recovered)	1884.8 (934.5, 3708.2)	<0.01	47.4 (−0.7, 118.9)	0.05
Vaccine Brand (Moderna vs. Pfizer-BioNTech)	−29.6 (−72.5, 80.1)	0.46	−34 (−59.1, 6.4)	0.09
log2 serum PFOS concentration	−0.1 (−42.7, 29.6)	0.72	0.3 (−30, 94)	0.31
Visit 3 vs. baseline	67965.9 (18340.8, 251135.1)	<0.01		
Visit 3 × log_2_ serum PFOS concentration	20.2 (−25.9, 66.4)	0.39		
Days from first vaccine			−0.7 (−3.4, 1.4)	0.44
Days from first vaccine × log_2_ serum PFOS concentration			−0.3 (−1, 1)	0.41

*GEE* generalized estimating equation, *AUC* area under the curve. *CI* confidence interval. *PFOA* Perfluorooctanoic acid (top), *PFOS* Perfluorooctanesulfonic acid (bottom).

^a^187 individuals with at least one anti-S antibody AUC in phase 1 were included in analysis.

^b^215 individuals with at least one anti-S antibody AUC in phase 2 were included in analysis.

### Waning anti-S antibody AUC over time from visit 3 to visit 4 (by PFAS concentration)

We assessed how anti-S antibody AUC changed over time from visit 3 to visit 4 (or, from day 42 through day 103), by calculating the change (delta) in log_2_ anti-S antibody AUC from visit 3 to visit 4 and used this as the outcome in a linear regression model. Neither PFOS, PFOA nor any other PFAS examined was found to impact the change in anti-S antibody AUC from visit 3 and visit 4 nor was prior COVID-19 infection status (all *p*-values > 0.05, see Table 4 (plus Supplementary Table S5) and Fig. 3B).

We also employed GEE model to examine the longitudinal change of AUC level from 42 days through 103 days after first vaccine. None of the PFAS examined were associated with changes in anti-S antibody AUCs during this post-vaccination phase (from 40 days to 103 days after first vaccine dose) (see Table 5 for PFOA and PFOS and Supplementary Table S4 for all other PFAS), all *p* values > 0.05. A trend of higher AUC among individuals with prior infection appeared during this phase but failed to meet criterion for significance (see Table 5). Increasing age was associated with a lower log_2_ anti-S antibody AUC by −0.04 units (95% CI: −0.06,−0.02) for the model with PFOA and PFOS. A waning effect, as indicated here by a slightly negative ‘days from first vaccine’ by ‘log_2_ serum PFOA concentration’ interaction term failed to meet criterion for significance. No effect of any PFAS was found on this phase of anti-S antibody AUC (all *p* values > 0.05) (see Table 5).

### PFAS mixture effects

Using the calculated change in log_2_ transformed anti-S antibody AUCs from baseline to visit 3, and visit 3 to visit 4 as outcomes, WQS and BKMR approaches were employed to assess the contribution of PFAS mixture exposures (see Supplemental Fig. S5 and Supplementary Table S6). The PFAS mixture was not associated with changes in anti-S antibody AUCs using these approaches.

## Discussion

In a population of adolescents and adults with a history of elevated PFAS exposure, we did not find an association between serum PFAS concentrations and antibody response to vaccination against COVID-19, despite prior indications of such effects in relation to routine childhood vaccine response. The 95th percentile and geometric mean of PFOS and PFOA serum concentrations among our participants were well above those of the general US population [34] and we described 11 unique PFAS that were found in over 60% of study participants. A population with a history of elevated PFAS exposure is expected to be the most likely to show adverse effects if such effects occur. Our results show that serum PFAS concentrations were not associated with antibody response to mRNA vaccination against COVID-19 in this highly-exposed adolescent and adult population and therefore we may expect a similar finding among the general population and those for whom PFAS serum concentrations are lower, although more research is needed to confirm these findings in other populations and exposure contexts. Although our population was recruited relatively early in the COVID-19 pandemic, which spanned a wide range of weekly case rates, and likely represented largely or exclusively cases of the original variant, we would not expect that the incidence rate of COVID-19 or the predominant variants during the time of our study would have altered the results of our study, since our design focused on the relationship between PFAS serum concentrations and antibody response to vaccination while controlling for prior infection.

Environmental contamination has long been associated with poor health and worsened disease outcomes among affected populations, and emerging evidence suggests COVID-19 is no exception [35–38]. Exposure to PFAS is likely one, among many, relevant environmental contaminants potentially influencing disease outcome and health trajectory. PFAS exposure, particularly PFOS and PFOA, may indeed directly impair immune response to vaccination, particularly among children. Experimental models have demonstrated a link between PFAS administration and suppression of antigen-specific antibody responses in animals [39–46] as have epidemiologic designs measuring childhood morbidity [47–53] and antigen-specific antibody concentrations following routine childhood immunization [22, 25, 49, 54, 55]. Animal studies [39, 40, 56, 57] have shown PFAS (specifically PFOS and PFOA) significantly suppresses the T cell-dependent antibody response (TDAR), which, although not specifying a mechanism of action, is widely considered a sensitive functional assay for evaluating immunosuppression [39, 58]. Moreover, there is some evidence that the immunotoxic effects of PFAS occur among animal models and highly exposed humans at comparable serum concentrations [59]. However, two human studies [25, 26] have failed to report associations *among adults* between PFAS exposure and antibody response (not specific to COVID-19) and our results align with these findings.

Thus far, research on the potential effect of PFAS on COVID-19 has consisted of limited attempts to relate PFAS blood concentrations to COVID-19 severity [60] or mortality [61]. While neither study attempted to examine vaccine responses, the rationale for the studies was a concern that higher levels of PFAS would impair the immunologic response to infection and thus increase susceptibility to severe [60] or fatal illness [61]. While these are the important endpoints for assessing the public health impact of PFAS on COVID-19, the data resources available to those investigators were limited for drawing any causal inferences, consisting of serum samples from COVID-19-infected biobank participants in Denmark [60] and ecologic information on PFAS blood concentrations and mortality in the Veneto Region of Italy [61]. The precision and specificity of studying individual PFAS blood concentrations and response to vaccine, as we have reported here, is more informative for understanding the potential adverse effects of PFAS on immunologic defenses to COVID-19. A recently published study [28] has described antibody response to COVID-19 vaccines among industrial workers with PFAS exposure and reported small associations between antibody levels and PFAS concentrations. It will likely be relevant to understand what impact the differences between that population and ours has on the results observed. The population described in the Porter et al. (2022) paper was generally younger than the population described here, with higher PFAS blood concentrations and higher rates of self-reported prior COVID-19 infection compared with our population. The authors also point out that all reported confidence intervals included zero in their analyses.

Overall, we did not observe significant differences in anti-S antibody AUC by serum PFAS concentration in our study population despite a thorough evaluation of that possibility. Specifically, we did not find (1) an effect of serum PFAS concentration on peak anti-S antibody AUC post-vaccination, (2) an effect of serum PFAS concentration on the change in anti-S antibody AUC during the initial increase period following vaccination, or (3) an effect of serum PFAS concentration on anti-S antibody AUC waning over time. We used a variety of approaches to arrive at this conclusion, including examining each PFAS alone (linear regression and GEE models) and in combination (WQS and BKMR models), and we examined the data longitudinally using repeated measures of anti-S antibody AUC and cross-sectionally using the change in anti-S antibody AUC from baseline to visit 3 (days 42–68 after first vaccine dose) as well as the change in anti-S antibody AUC between visit 3 and 4 (days 70–103 after first vaccine dose). Results from all approaches taken together provide consistent evidence indicating that serum PFAS concentrations had little or no impact on vaccine response (anti-S antibody AUC) among this population. One possible contributing factor to this could be that the mRNA vaccine has been shown to have a more general adjuvating effect compared with traditional vaccine products. The absence of an impact by PFAS exposure may be influenced by the fact that mRNA vaccines lead to durable CD4^+^ T cell response and superior humoral immunity [62].

The PFOS and PFOA serum concentrations reported here describe a population that has been highly impacted by exposure to PFOS and PFOA, in particular. Not only did our study population, on average, have higher blood concentrations of PFOS and PFOA compared to the national average, but they are similarly exposed as other populations where associations to vaccine response have been demonstrated. For example, other studies describing reduced antibody concentrations following non-COVID-19 vaccination have also reported serum PFOS and PFOA concentrations within the range or below those of our study population [22–24].

The ELISA assay used here successfully quantified the anti-S antibody response. Contributing to this conclusion is the evidence that our method for quantifying anti-S antibodies was successful and captured the expected initial rise and gradual plateau of anti-S antibodies in the months following vaccination. Moreover, the pattern and magnitude of response was similar to those seen in Moderna clinical trials [31, 32]. We found that age and prior COVID-19 infection status were important determinants of vaccine response, which was also expected. Further, based on power calculations for the linear regression model, to detect an effect of serum PFAS on antibody response (Partial R-square 6% and type I error as 0.05), we would need 99 observations to achieve 70% power. The sample size achieved in our study was well above this and permits adequate statistical power.

In addition to using a quantitative (as opposed to qualitative) approach to understanding antibody response, we also measured many PFAS (39 analytes) and were able to examine the potential impact of PFAS beyond those most commonly described in the literature (e.g., legacy PFAS like PFOS and PFOA). We identified 11 unique PFAS detected in more than 60% of participants and evaluated these both alone and as a mixture. We specifically attempted to address the issue (and expectation) of correlated exposure to many individual PFAS. Humans rarely, if ever, encounter exposure to a single PFAS and often there is correlation among the PFAS to which they are exposed. We noted the correlations among several PFAS and included two statistical approaches that permit analysis of exposure to mixtures. WQS regression is a constrained regression approach that was designed to estimate the effect of a mixture of correlated chemicals. BKMR uses a kernel function to estimate the multivariable exposure-response function in a flexible way that allows for nonlinear and non-additive effects, while adjusting for covariates including potential confounding factors. Also, BKMR employs a hierarchical variable selection approach that addresses the issue of multicollinearity by first classifying highly correlated exposures into groups, and then simultaneously conducting variable selection on the groups of correlated exposures as well as on the individual exposures within each group.

### Limitations

The impact of the timing of vaccination during the lifespan, the follow-up window observed or the timing of PFAS exposure could each contribute to the differences observed here compared to earlier studies where reduced antibody response to vaccination has been described among individuals with elevated PFAS exposure. The most robust vaccine response effects have been reported in children with a history of prenatal or early life PFAS exposure where antibody response was observed to be reduced as a function of blood PFAS concentration many years following initial vaccinations [22]. Our participants were adolescents and adults when first administered a novel vaccine and they were followed for the first 3.5 months following their first dose of a novel vaccine, and most of our data correspond to a period 40 days to 100 days after vaccination. It is possible that the design of this study missed the window where PFAS exerted an impact on antibody response. Perhaps it was very early following administration of the first vaccine, in the first days or weeks of the immune response. A study in adults found PFAS affected the rate of increase in antibody response after a diphtheria-tetanus booster between days 4 and 10 after vaccine [23]. The clinical significance of an immune response constrained to just this window, and disappearing soon after, is likely marginal. It is also possible that PFAS could impact the durability of the antibody response and longer follow-up could reveal differences in antibody levels by serum PFAS concentration. Our own follow-up studies in this population are ongoing, including data collection anchored to booster doses, and long-term follow-up among these participants may address this limitation. Or it might be that PFAS has relatively subtle impacts on immunogenicity among adolescents and adults, and the mRNA vaccines against COVID-19 examined here are too immunogenic for those impacts to be seen.

While our method was useful for quantifying the anti-S antibody response to vaccine, the study did not include infection as an outcome and was not a study of vaccine efficacy. Binding antibodies, as measured by the ELISA assay used in this study, have been shown to correlate well with neutralizing antibody to the ancestral SARS-CoV-2 which is the basis for the vaccines studied here [28]. However, the correlation between binding and neutralizing antibodies against to SARS-CoV-2 variants is unclear overall, and likely particularly low for Omicron-descendent viruses. While studies have demonstrated that seropositivity after infection and vaccination provide protection against infection and severe COVID-19, a protective SARS-CoV-2 antibody titer has not been established especially in light of the ongoing introduction of novel variants. Additionally, humoral immunity is but one component of immune response to and protection from infection. How our antibody measurements relate to protection against outcomes such as infection or disease severity was not evaluated in this study.

As part of this study, we did not include PFAS source attribution aims and did not explore other mechanisms that might impact circulating serum PFAS concentrations (e.g., kidney function). Therefore, we are not able to directly speak to the influence that factors like kidney function or any particular environmental PFAS source might have had on serum PFAS concentrations. Questions related to how such factors might influence the relationship between PFAS and antibody response to vaccine is an area for future research. Finally, the mean age of our population was over 50 years old and may not represent antibody response among younger populations. The age of our participants reflects the availability of COVID-19 vaccines during the time we enrolled for this study, as the vaccines became available for younger groups during and after our enrollment period.

### Conclusions

Many factors contribute to variability in individual response to immunizations, including age, medications, and underlying health conditions. Whether exposure to environmental chemicals, like PFAS, contribute to this variability in adults is an important question. We examined this question within the context of immunization against COVID-19. Although we describe a population of adolescents and adults highly impacted by environmental PFAS contamination, and for whom exposure was primarily via drinking water, our results do not support the hypothesis that higher serum PFAS concentration reduces antibody response to mRNA vaccines against COVID-19. Future studies are needed to clarify the impact of age of vaccination (early life vs. adulthood vaccination), period of PFAS exposure (gestational/early life vs. later in life) and timeframe of when the effects of PFAS might manifest in antibody response to vaccination (months vs. years following vaccination).

### Supplementary Information

Supplementary materials

Reporting Checklist

## Data Availability

The data used during the current study are available from the corresponding author on reasonable request and consistent with MDHHS policy and procedures.
