# Supplementary material for: Immune response to COVID-19 vaccination in a population with a history of elevated exposure to per- and polyfluoroalkyl substances (PFAS) through drinking water
Source: J Expo Sci Environ Epidemiol. 2023 Jun 19;33(5):725–36. doi: 10.1038/s41370-023-00564-8 (PMC10541329; doi:10.1038/s41370-023-00564-8)
Supplement: Supplementary file 1 — Supplementary materials [file 41370_2023_564_MOESM1_ESM.docx]

**Supplemental Table S1.** Individual PFAS measured in serum of participants and their corresponding limit of quantification (LOQ).

| Abbreviation | Name | CAS Number | MDHHS Method LOQ (μg/L) | Detection Rate (%) |
| --- | --- | --- | --- | --- |
| 11Cl-PF3OUdS | 11-chloroeicosafluoro-3-oxaundecane-1-sulfonate | 763051-92-9 | 0.0237 | 0% |
| 3:3 FTCA | 2H,2H,3H,3H-perfluorohexanoic acid (3-perfluoropropyl propanoic acid) | 356-02-5 | 0.0224 | 0% |
| 4:2 FTS | 1H, 1H, 2H, 2H, perfluorohexane sulfonic acid | 757124-72-4 | 0.0087 | 0% |
| 5:3 FTCA | 2H,2H,3H,3H-Perfluorooctanoic acid (3-perfluoropentyl propanoic acid) | 914637-49-3 | 0.0241 | 5.31% |
| 6:2 FTS | 1H, 1H, 2H, 2H, perfluorooctane sulfonic acid | 27619-97-2 | 0.0229 | 0% |
| 7:3 FTCA | 2H,2H,3H,3H-Perfluorodecanoic acid (3-perfluoroheptyl propanoic acid) | 812-70-4 | 0.0242 | 1.77% |
| 8:2 FTS | 1H, 1H, 2H, 2H, perfluorodecane sulfonic acid | 39108-34-4 | 0.0180 | 8.85% |
| 9Cl-PF3ONS | 9-chlorohexadecafluoro-3-oxanonane-1-sulfonate | 756426-58-1 | 0.0201 | 13.27% |
| ADONA | Dodecafluoro-3H-4,8-dioxanonanoate *or* 4,8-dioxa-3H-perfluorononanoic acid *(ADONA)* | 919005-14-4 | 0.0182 | 0% |
| EtFOSAA | N-Ethylperfluorooctane sulfonamidoacetic acid | 2991-50-6 | 0.0227 | 19.91% |
| HFPO-DA | Hexafluoropropylene oxide dimer acid *(GenX* *)* | 13252-13-6 | 0.0083 | 0% |
| NFDHA | Nonafluoro-3,6-dioxaheptanoic acid | 151772-58-6 | 0.0239 | 0% |
| MeFOSAA | N-Methylperfluorooctane sulfonamidoacetic acid | 2355-31-9 | 0.0132 | 93.36% |
| PFBA | Perfluorobutanoic acid | 375-22-4 | 0.0176 | 57.52% |
| PFBS | Perfluorobutanesulfonic acid | 375-73-5 | 0.0125 | 38.94% |
| PFBSA | Perfluorobutanesulfonamide | 30334-69-1 | 0.0189 | 0% |
| PFDA | Perfluorodecanoic acid | 335-76-2 | 0.0159 | 96.90% |
| PFDoA | Perfluorododecanoic acid | 307-55-1 | 0.0145 | 18.58% |
| PFDS | Perfluorodecanesulfonic acid | 335-77-3 | 0.0219 | 3.54% |
| PFEESA | Perfluoro (2-ethoxyethane) sulfonic acid | 113507-82-7 | 0.0178 | 0.44% |
| PFHpS | Perfluoroheptanesulfonic acid | 375-92-8 | 0.0230 | 96.90% |
| PFHxA | Perfluorohexanoic acid | 307-24-4 | 0.0113 | 0.44% |
| PFHxS (total) | Perfluorohexanesulfonic acid (branched and linear) | 355-46-4 |  |  |
| L-PFHxS | Perfluorohexanesulfonic acid (linear) |  | 0.0241 | 99.56% |
| Br-PFHxS | Perfluorohexanesulfonic acid (branched) |  | 0.0234 | 22.57% |
| PFHxSA | Perfluorohexanesulfonamide | 41997-13-1 | 0.0241 | 0% |
| PFMBA | Perfluoro-4-methoxybutanoic acid | 863090-89-5 | 0.0233 | 0% |
| PFMPA | Perfluoro-3-methoxypropanoic acid | 377-73-1 | 0.0221 | 0% |
| PFNA | Perfluorononanoic acid | 375-95-1 | 0.0235 | 99.12% |
| PFNS | Perfluorononanesulfonic acid | 68259-12-1 | 0.0198 | 0% |
| PFOA (total) | Perfluorooctanoic acid (branched and linear) | 335-67-1 |  | 100% |
| L-PFOA | Perfluorooctanoic acid (linear) |  | 0.0145 | 100% |
| Br-PFOA | Perfluorooctanoic acid (branched) |  | 0.0145 | 26.99% |
| PFOS (total) | Perfluorooctanesulfonic acid (branched and linear) | 1763-23-1 |  | 100% |
| L-PFOS | Perfluorooctanesulfonic acid (linear) |  | 0.0231 | 99.56% |
| Br-PFOS | Perfluorooctanesulfonic acid (branched) |  | 0.0239 | 100% |
| PFOSA | Perfluorooctanesulfonamide | 754-91-6 | 0.0192 | 0% |
| PFPeA | Perfluoropentanoic acid | 2706-90-3 | 0.0160 | 0% |
| PFPeS | Perfluoropentanesulfonic acid | 2706-91-4 | 0.0149 | 73.01% |
| PFPrS | Perfluoropropanesulfonic acid | 423-41-6 | 0.0205 | 0% |
| PFTeA | Perfluorotetradecanoic acid | 376-06-7 | 0.0149 | 0.88% |
| PFTriA | Perfluorotridecanoic acid | 72629-94-8 | 0.0236 | 8.85% |
| PFUnA | Perfluoroundecanoic acid | 2058-94-8 | 0.0185 | 82.30% |
| PFHpA | Perfluoroheptanoic acid | 375-85-9 | 0.0167 | 60.18% |
| PFECHS | Perfluoroethylcyclohexane sulfonate | 646-83-3 | 0.0223 | 82.30% |

# *Table notes*. MDHHS = Michigan Department of Health and Human Services; “L-“= linear isomer; “Br-“ = branched isomer. “Total” refers to the sum of Br and L isomers and is reported for PFHxS, PFOA and PFOS.

**Supplement Table S2.** Descriptive statistics for the 11 PFAS (µg/L) that were detected in at least 60% of participants (N=226).

| **PFAS** | **Geometric Mean (GSD)** | **25^th^ percentile** | **50^th^ percentile** | **75^th^ percentile** | **95^th^ percentile** |
| --- | --- | --- | --- | --- | --- |
| PFOS | 10.49 (3.22) | 5.25 | 9.09 | 21.79 | 79.92 |
| PFOA | 3.90 (4.90) | 1.06 | 2.43 | 14.94 | 61.81 |
| PFHxS | 1.53 (3.72) | 0.68 | 1.48 | 3.34 | 11.11 |
| PFHpS | 0.38 (4.22) | 0.14 | 0.34 | 0.99 | 3.84 |
| PFNA | 0.36 (2.17) | 0.24 | 0.38 | 0.57 | 1.13 |
| MeFOSAA | 0.12 (2.76) | 0.06 | 0.09 | 0.24 | 0.95 |
| PFDA | 0.09 (2.63) | 0.06 | 0.1 | 0.18 | 0.37 |
| PFPeS | 0.04 (2.41) | 0.01 | 0.03 | 0.06 | 0.26 |
| PFUNA | 0.04 (2.72) | 0.01 | 0.05 | 0.09 | 0.19 |
| PFecHS | 0.03 (3.45) | 0.03 | 0.04 | 0.06 | 0.12 |
| PFHpA | 0.02 (2.77) | 0.01 | 0.01 | 0.05 | 0.1 |

*Table notes*. GSD= geometric standard deviation. All PFAS are log_2_ transformed. See **Supplemental Table S1** for full name and CAS# of each PFAS listed here.

**Appendix Table S3.** Linear regression results for change of anti-S antibody AUC at visit 3 and visit 4 for all PFAS detected in >60% of participants.

|  | Log_2_ anti-S antibody AUC at visit 3 | | | Log_2_ anti-S antibody AUC at visit 4 | |
| --- | --- | --- | --- | --- | --- |
|  | Percentage Change (95% CI) | P-value | | Percentage Change (95% CI) | P-value |
| MeFOSAA | -17.8(-47.5,12) | 0.24 | | -14.2(-36.1,7.7) | 0.21 |
| Age | -2.5(-4.3,-0.8) | 0.01 | | -3(-4.3,-1.7) | <0.01 |
| Female vs. male | -25.5(-59.3,36.3) | 0.34 | | 11.4(-28.9,74.5) | 0.64 |
| Geographical site | -36.7(-66.2,18.5) | 0.16 | | -9.5(-42.1,41.4) | 0.66 |
| COVID-19 history (recovered vs. naïve) | 92.6(3.3,259) | 0.04 | | 24.1(-21.3,95.6) | 0.35 |
| Pfizer-BioNTech | -15.1(-63.6,97.7) | 0.7 | | -25.8(-57.5,29.8) | 0.3 |
| PFDA | -3.1(-35.7,29.6) | 0.85 | | 5.2(-18.3,28.7) | 0.67 |
| Age | -2.7(-4.4,-0.9) | <0.01 | | -3.1(-4.4,-1.9) | <0.01 |
| Female vs. male | -24.6(-59.3,39.4) | 0.37 | | 13.5(-28,78.9) | 0.59 |
| Geographical site | -39.6(-68.1,14.1) | 0.12 | | -13.9(-45.5,35.9) | 0.52 |
| COVID-19 history (recovered vs. naïve) | 90.8(1.2,259.8) | 0.05 | | 23.2(-22.2,94.9) | 0.37 |
| Pfizer-BioNTech | -12.9(-62.9,104.8) | 0.75 | | -26(-57.9,29.9) | 0.3 |
| PFHpA | 33.6(2.4,64.7) | 0.14 | | 9(-12.8,30.8) | 0.42 |
| Age | -2.9(-4.6,-1.1) | <0.01 | | -3.2(-4.4,-1.9) | <0.01 |
| Female vs. male | -21.9(-57,41.8) | 0.42 | | 11.1(-29.2,74.2) | 0.65 |
| Geographical site | -41.7(-68.4,7.6) | 0.09 | | -12.4(-43.9,36.8) | 0.56 |
| COVID-19 history (recovered vs. naïve) | 95.2(5.4,261.4) | 0.04 | | 22.3(-22.4,92.9) | 0.39 |
| Pfizer-BioNTech | -15.1(-63.2,95.8) | 0.7 | | -25.4(-57.4,30.7) | 0.31 |
| PFHpS | 10.7(-11.2,32.7) | 0.34 | | 0.8(-16.2,17.8) | 0.93 |
| Age | -2.8(-4.6,-1) | <0.01 | | -3.1(-4.4,-1.8) | <0.01 |
| Female vs. male | -19(-56.3,50.2) | 0.5 | | 12.4(-29.3,78.5) | 0.62 |
| Geographical site | -35(-65.9,23.7) | 0.19 | | -11.5(-44.7,41.7) | 0.61 |
| COVID-19 history (recovered vs. naïve) | 91.5(2.6,257.5) | 0.04 | | 21.6(-23,92) | 0.4 |
| Pfizer-BioNTech | -19.7(-66,89.7) | 0.62 | | -25.3(-57.4,31) | 0.31 |
| PFNA | -5.7(-44.1,32.6) | 0.77 | | 1.5(-27.8,30.9) | 0.92 |
| Age | -2.6(-4.4,-0.8) | 0.01 | | -3.1(-4.4,-1.8) | <0.01 |
| Female vs. male | -24.7(-59.1,38.6) | 0.36 | | 12.1(-28.8,76.6) | 0.62 |
| Geographical site | -40.1(-67.8,11.7) | 0.11 | | -12.2(-43.8,37.3) | 0.57 |
| COVID-19 history (recovered vs. naïve) | 91.3(2.1,258.3) | 0.04 | | 21.8(-22.8,92.2) | 0.4 |
| Pfizer-BioNTech | -12.2(-62.7,106.8) | 0.77 | | -25.4(-57.5,31) | 0.31 |
| PFPeS | 13.8(-9.2,36.7) | 0.24 | 0.1(-17.8,18) | | 0.99 |
| Age | -2.7(-4.4,-0.9) | <0.01 | -3.1(-4.4,-1.8) | | <0.01 |
| Female vs. male | -23.8(-58.3,39.2) | 0.38 | 11.8(-28.8,75.7) | | 0.63 |
| Geographical site | -41.5(-68.5,8.7) | 0.09 | -12.1(-43.8,37.4) | | 0.57 |
| COVID-19 history (recovered vs. naïve) | 96.1(5.1,265.9) | 0.04 | 21.7(-22.9,92.1) | | 0.4 |
| Pfizer-BioNTech | -16.6(-64.2,94.6) | 0.68 | -25.2(-57.3,31.1) | | 0.31 |
| PFUNA | -14.8(-46.3,16.7) | 0.36 | -10.7(-33.7,12.2) | | 0.36 |
| Age | -2.6(-4.3,-0.7) | 0.01 | -3(-4.3,-1.7) | | <0.01 |
| Female vs. male | -27(-60.3,34.5) | 0.31 | 9(-30.7,71.5) | | 0.71 |
| Geographical site | -37.8(-66.7,16.4) | 0.14 | -9(-42,43) | | 0.68 |
| COVID-19 history (recovered vs. naïve) | 89.3(1.3,253.8) | 0.05 | 19.4(-24.4,88.6) | | 0.45 |
| Pfizer-BioNTech | -9.9(-61.6,111.2) | 0.81 | -23.5(-56.4,34.2) | | 0.35 |
| PFECHS | -9.7(-45.4,26) | 0.59 | -5.2(-33,22.6) | | 0.72 |
| Age | -2.6(-4.4,-0.7) | 0.01 | -3(-4.4,-1.7) | | <0.01 |
| Female vs. male | -26.8(-60.8,36.4) | 0.33 | 9.7(-30.9,74.1) | | 0.7 |
| Geographical site | -37.9(-67.2,17.6) | 0.15 | -10.2(-43.3,42.1) | | 0.65 |
| COVID-19 history (recovered vs. naïve) | 92.3(2.9,259.5) | 0.04 | 21.4(-23.1,91.6) | | 0.41 |
| Pfizer-BioNTech | -10.5(-62.1,111.3) | 0.8 | -24.4(-57,32.8) | | 0.33 |
| PFHxS | 14.9(-7.7,37.4) | 0.2 | -3.3(-21,14.5) | | 0.72 |
| Age | -2.8(-4.6,-1) | <0.01 | -3.1(-4.4,-1.8) | | <0.01 |
| Female vs. male | -16.4(-55,55.3) | 0.57 | 9.8(-30.8,74.2) | | 0.69 |
| Geographical site | -38.9(-67.1,13.5) | 0.12 | -13.2(-44.7,36.4) | | 0.54 |
| COVID-19 history (recovered vs. naïve) | 93.7(4,260.9) | 0.04 | 22.1(-22.6,92.8) | | 0.39 |
| Pfizer-BioNTech | -22.6(-67.2,83) | 0.56 | -24.4(-57,32.9) | | 0.33 |

*Table notes.* AUC = area under the curve. CI = confidence interval. PFOA = Perfluorooctanoic acid (top). PFOS = Perfluorooctanesulfonic acid (bottom).

**Supplemental Table S4.** GEE regression results with interaction of serum PFAS concentration (for PFAS detected in 60% or more of participants) and days from first vaccine to explain outcome in log_2_ anti-S antibody AUC.

|  | Day 0 to day 68 after first vaccine^1^  (initial increase phase) | | Day 42 to day 103 after first vaccine^2^  (waning phase) | | | | | | | |
| --- | --- | --- | --- | --- | --- | --- | --- | --- | --- | --- |
|  | **Percentage Change (95% CI)** | **P-value** | **Percentage Change (95% CI)** | | **P-value** | | |  |  |  |
| Age | -1.3(-3.2,0.7) | 0.21 | -2.7(-3.7,-1.7) | | <0.01 | | |  |  |  |
| Female vs. male | -16.3(-56.7,61.6) | 0.60 | -4.3(-33.7,38) | | 0.81 | | |  |  |  |
| Geographical site | -23.7(-59.9,45.2) | 0.41 | -22.1(-45.2,10.7) | | 0.20 | | |  |  |  |
| COVID-19 history (recovered vs. naïve) | 1798.4(889.4,3542.4) | <0.01 | 51.3(5.9,116.4) | | 0.02 | | |  |  |  |
| Moderna vs. Pfizer-BioNTech | -25.6(-71,90.9) | 0.54 | -22.5(-51.6,24.1) | | 0.29 | | |  |  |  |
| log_2_ serum MeFOSAA concentration | 0.3(-22.9,84.3) | 0.26 | -21.8(-122.8,79.3) | | 0.67 | | |  |  |  |
| Visit 3 vs. baseline | 35347.9(8695.1,142769.4) | <0.01 |  | |  | | |  |  |  |
| Visit 3 × log_2_ serum MeFOSAA concentration | -55.2(-117.4,7) | 0.08 |  | |  | | |  |  |  |
| Days from first vaccine |  |  | -1.3(-4.6,2.2) | | 0.47 | | |  |  |  |
| Days from first vaccine  × log_2_ serum MeFOSAA concentration |  |  | 0.1(-1.3,1.5) | | 0.89 | | |  |  |  |
| Age | -1.2(-3.1,0.7) | 0.22 | -2.8(-3.8,-1.7) | | <0.01 | | |  |  |  |
| Female vs. male | -17.6(-57.3,59) | 0.56 | -3(-33.1,40.5) | | 0.87 | | |  |  |  |
| Geographical site | -24.6(-60.1,42.5) | 0.38 | -25.1(-47.8,7.5) | | 0.12 | | |  |  |  |
| COVID-19 history (recovered vs. naïve) | 1877.8(931.3,3693.2) | <0.01 | 49.9(4.5,114.8) | | 0.03 | | |  |  |  |
| Moderna vs. Pfizer-BioNTech | -26.7(-70.8,84.4) | 0.51 | -21.1(-50.7,26.5) | | 0.33 | | |  |  |  |
| log_2_ serum PFNA concentration | 0(-59.6,55.2) | 0.94 | -3.1(-129.7,123.4) | | 0.96 | | |  |  |  |
| Visit 3 vs. baseline | 106013.1(39292.2,285742.9) | <0.01 |  | |  | | |  |  |  |
| Visit 3 × log_2_ serum PFNA concentration | -2.3(-73.6,68.9) | 0.95 |  | |  | | |  |  |  |
| Days from first vaccine |  |  | -1.4(-3.5,0.7) | | 0.19 | | |  |  |  |
| Days from first vaccine  × log_2_ serum PFNA concentration |  |  | 0(-1.6,1.7) | | 0.97 | | |  |  |  |
| Age | -1.4(-3.3,0.6) | 0.17 | -2.9(-3.9,-1.9) | | <0.01 | | |  |  |  |
| Female vs. male | -16.9(-56.9,60.3) | 0.58 | -3(-32.7,40) | | 0.87 | | |  |  |  |
| Geographical site | -25.8(-60.8,40.7) | 0.36 | -27.2(-49.2,4.4) | | 0.08 | | |  |  |  |
| COVID-19 history (recovered vs. naïve) | 1886(945,3674.2) | <0.01 | 50.5(5.5,114.8) | | 0.02 | | |  |  |  |
| Moderna vs. Pfizer-BioNTech | -27.4(-71.5,84.9) | 0.5 | -21.6(-50.6,24.6) | | 0.30 | | |  |  |  |
| log_2_ serum PFHpA concentration | 0.1(-43.7,57.4) | 0.79 | 70.1(-14.9,155.1) | | 0.11 | | |  |  |  |
| Visit 3 vs. baseline | 273675(21138.2,3529045.1) | <0.01 |  | |  | | |  |  |  |
| Visit 3 × log_2_ serum PFHpA concentration | 22.8(-40.1,85.7) | 0.48 | |  | |  | | |  |  |
| Days from first vaccine |  |  | | -4.1(-8.3,0.2) | | 0.06 | | |  |  |
| Days from first vaccine  × log_2_ serum PFHpA concentration |  |  | | -0.7(-1.8,0.5) | | 0.26 | | |  |  |
| Age | -1.2(-3.2,0.8) | 0.23 | | -2.8(-3.9,-1.8) | | <0.01 | | |  |  |
| Female vs. male | -16.1(-57,63.5) | 0.61 | | -0.1(-30.8,44.3) | | 0.99 | | |  |  |
| Geographical site | -24.1(-60.9,47.3) | 0.42 | | -22.9(-46.7,11.6) | | 0.17 | | |  |  |
| COVID-19 history (recovered vs. naïve) | 1869.9(932.5,3658.7) | <0.01 | | 51.6(5.2,118.3) | | 0.03 | | |  |  |
| Moderna vs. Pfizer-BioNTech | -28.5(-71.8,80.8) | 0.48 | | -23.7(-52.6,22.9) | | 0.27 | | |  |  |
| log_2_ serum PFHpS concentration | -0.1(-43,32.3) | 0.78 | | 39(-15,93) | | 0.16 | | |  |  |
| Visit 3 vs. baseline | 122496.8(58897.9,254654.4) | <0.01 | |  | |  | | |  |  |
| Visit 3 × log_2_ serum PFHpS concentration | 12.6(-31.3,56.4) | 0.57 | |  | |  | | |  |  |
| Days from first vaccine |  |  | | -1.9(-3.3,-0.6) | | 0.01 | | |  |  |
| Days from first vaccine  × log_2_ serum PFHpS concentration |  |  | | -0.5(-1.2,0.3) | | 0.19 | | |  |  |
| Age | -1.2(-3.2,0.8) | 0.22 | | -2.8(-3.8,-1.7) | | <0.01 | | |  |  |
| Female vs. male | -19.1(-58.2,56.7) | 0.53 | | -2.3(-33.1,42.5) | | 0.90 | | |  |  |
| Geographical site | -23.2(-59.5,45.7) | 0.42 | | -26.5(-48.7,5.3) | | 0.09 | | |  |  |
| COVID-19 history (recovered vs. naïve) | 1842.4(910.1,3635) | <0.01 | | 51.1(4.8,117.7) | | 0.03 | | |  |  |
| Moderna vs. Pfizer-BioNTech | -21.5(-68.8,97.1) | 0.61 | | -22.3(-51.7,24.8) | | 0.29 | | |  |  |
| log_2_ serum PFDA concentration | -0.4(-80.6,5.8) | 0.09 | | -14.6(-96.8,67.6) | | 0.73 | | |  |  |
| Visit 3 vs. baseline | 342545.6(74552,1572610.5) | <0.01 | |  | |  | | |  |  |
| Visit 3 × log_2_ serum PFDA concentration | 47.7(-5.2,100.7) | 0.08 | |  | |  | | |  |  |
| Days from first vaccine |  |  | | -0.9(-3.8,2.2) | | 0.57 | | |  |  |
| Days from first vaccine  × log_2_ serum PFDA concentration |  |  | | 0.3(-0.8,1.3) | | 0.65 | | |  |  |
| Age | -1.2(-3.1,0.8) | 0.23 | | -2.8(-3.8,-1.7) | | <0.01 | | |  |  |
| Female vs. male | -18.8(-57.9,56.6) | 0.53 | | -2.8(-32.9,40.6) | | 0.88 | | |  |  |
| Geographical site | -24.3(-59.9,43.1) | 0.39 | | -26(-48.4,6.3) | | 0.10 | | |  |  |
| COVID-19 history (recovered vs. naïve) | 1877.4(930.5,3694.3) | <0.01 | | 51.6(5.8,117.3) | | 0.02 | | |  |  |
| Moderna vs. Pfizer-BioNTech | -28.3(-71.6,81.3) | 0.48 | | -21.9(-51.2,24.9) | | 0.30 | | |  |  |
| log_2_ serum PFPeS concentration | -0.2(-60.8,26.6) | 0.44 | | 35.9(-22.3,94.1) | | 0.23 | | |  |  |
| Visit 3 vs. baseline | 350532.2(59769.3,2053423.6) | <0.01 | |  | |  | | |  |  |
| Visit 3 × log_2_ serum PFPeS concentration | 34.3(-15.1,83.7) | 0.17 | |  | |  | | |  |  |
| Days from first vaccine |  |  | | -2.9(-5.5,-0.2) | | 0.04 | | |  |  |
| Days from first vaccine  × log_2_ serum PFPeS concentration |  |  | | -0.4(-1.2,0.4) | | | 0.29 | | |  |
| Age | -1.1(-3,1) | 0.3 | | -2.7(-3.7,-1.6) | | <0.01 | | |  |  |
| Female vs. male | -20.8(-58.7,52.1) | 0.48 | | -5.5(-34.9,37.2) | | 0.77 | | |  |  |
| Geographical site | -21.6(-58.5,47.9) | 0.45 | | -22.3(-45.9,11.5) | | 0.17 | | |  |  |
| COVID-19 history (recovered vs. naïve) | 1842.6(917.7,3608.3) | <0.01 | | 47.3(2.6,111.5) | | 0.04 | | |  |  |
| Moderna vs. Pfizer-BioNTech | -21.8(-69.9,103.6) | 0.62 | | -18.4(-49.3,31.3) | | 0.4 | | |  |  |
| log_2_ serum PFNA concentration | -0.2(-70.4,27.8) | 0.40 | | -11.8(-88.8,65.3) | | 0.76 | | |  |  |
| Visit 3 vs. baseline | 120488.9(13862.3,1041396.4) | <0.01 | |  | |  | | |  |  |
| Visit 3 × log_2_ serum PFUnA concentration | 3.7(-57,64.4) | 0.91 | |  | |  | | |  |  |
| Days from first vaccine |  |  | | -1.4(-5,2.3) | | 0.45 | | |  |  |
| Days from first vaccine  × log_2_ serum PFUnA concentration |  |  | | 0(-1,1) | | 0.99 | | |  |  |
| Age | -1.2(-3.2,0.8) | 0.24 | | -2.7(-3.7,-1.6) | | <0.01 | | |  |  |
| Female vs. male | -18.3(-58.5,61.1) | 0.56 | | -6(-35.6,37) | | 0.75 | | |  |  |
| Geographical site | -23.5(-60.4,47.8) | 0.43 | | -22.4(-46.2,11.8) | | 0.17 | | |  |  |
| COVID-19 history (recovered vs. naïve) | 1939(969.5,3787.4) | <0.01 | | 49.7(4.6,114) | | 0.03 | | |  |  |
| Moderna vs. Pfizer-BioNTech | -28.8(-70.9,74.5) | 0.46 | | -19.3(-49.6,29.5) | | 0.38 | | |  |  |
| log_2_ serum PFECHS concentration | 0.2(-43.9,76.7) | 0.59 | | -1.8(-103.6,99.9) | | 0.97 | | |  |  |
| Visit 3 vs. baseline | 29559.5(2548.6,332038.8) | <0.01 | |  | |  | | |  |  |
| Visit 3 × log_2_ serum PFECHS concentration | -39.2(-110.1,31.7) | 0.28 | |  | |  | | |  |  |
| Days from first vaccine |  |  | | -1.8(-6.6,3.3) | | 0.48 | | |  |  |
| Days from first vaccine  × log_2_ serum PFECHS concentration |  |  | | -0.1(-1.5,1.3) | | 0.89 | | |  |  |
| Age | -1.2(-3.1,0.8) | 0.25 | | -2.8(-3.8,-1.7) | | | <0.01 | | |  |
| Female vs. male | -16.7(-57.2,62.1) | 0.59 | | -0.2(-31.1,44.8) | | | 0.99 | | |  |
| Geographical site | -25.5(-60.7,41.2) | 0.37 | | -25.3(-47.6,6.5) | | | 0.1 | | |  |
| COVID-19 history (recovered vs. naïve) | 1855.7(922.9,3639.3) | <0.01 | | 52.3(5.9,119.2) | | | 0.02 | | |  |
| Moderna vs. Pfizer-BioNTech | -28.3(-71.9,82.9) | 0.49 | | -23.1(-52.2,23.8) | | | 0.28 | | |  |
| log_2_ serum PFHxS concentration | -0.1(-54.9,26.6) | 0.5 | | 49.9(-9.4,109.2) | | | 0.09 | | |  |
| Visit 3 vs. baseline | 96014.9(46519.4,198059.2) | <0.01 | |  | | |  | | |  |
| Visit 3 × log_2_ serum PFHxS concentration | 25.9(-21,72.8) | 0.28 | |  | | |  | | |  |
| Days from first vaccine |  |  | | -1.2(-2.4,0.1) | | | 0.08 | | |  |
| Days from first vaccine  × log_2_ serum PFHxS concentration |  |  | | -0.7(-1.5,0.2) | | | 0.11 | | |  |

*Table notes*. CI= confidence interval. All PFAS are log_2_ transformed. See **Supplemental Table S1** for full name and CAS# of each PFAS listed here.

**Appendix Table S5.** Linear regression results for change of anti-S antibody AUC from baseline to visit 3 and the change from visit 3 to visit 4 for all PFAS detected in >60% of participants.

|  | Log_2_ anti-S antibody AUC change from  day 0 to day 68 after first vaccine  (initial increase phase) | | Log_2_ anti-S antibody AUC change from  day 42 to day 103 after first vaccine  (waning phase) | |
| --- | --- | --- | --- | --- |
|  | Percentage Change (95% CI) | P-value | Percentage Change (95% CI) | P-value |
| MeFOSAA | -44.8(-120.5,31) | 0.25 | 20.5(-13.7,54.7) | 0.24 |
| Age | 0.9(-3.7,5.7) | 0.71 | -1.2(-3.2,0.9) | 0.28 |
| Female vs. male | -41.3(-86.7,159.6) | 0.48 | 73.8(-13.7,249.9) | 0.12 |
| Geographical site | -39.4(-87.1,183.8) | 0.53 | 46.7(-29.1,203.4) | 0.30 |
| COVID-19 history (recovered vs. naïve) | -98.9(-99.8,-95) | <0.001 | -47.2(-74.3,8.2) | 0.08 |
| Pfizer-BioNTech | 77.6(-76.5,1244) | 0.58 | 4.8(-61.9,188) | 0.93 |
| PFDA | 21.1(-59.6,101.9) | 0.61 | -0.1(-37.1,37) | 0.99 |
| Age | 0.5(-4,5.3) | 0.82 | -1(-3.1,1.1) | 0.33 |
| Female vs. male | -35.7(-86.2,200.4) | 0.58 | 71.5(-16,250.2) | 0.14 |
| Geographical site | -54(-89.9,109.4) | 0.32 | 59.5(-23.8,233.9) | 0.22 |
| COVID-19 history (recovered vs. naïve) | -98.9(-99.8,-94.5) | <0.001 | -47(-74.6,10.2) | 0.09 |
| Pfizer-BioNTech | 72.8(-78.5,1290.3) | 0.61 | -0.6(-64.1,175.5) | 0.99 |
| PFHpA | 52.9(-26,131.7) | 0.19 | -0.9(-38,36.1) | 0.96 |
| Age | 0(-4.5,4.8) | 0.99 | -1(-3.1,1.1) | 0.34 |
| Female vs. male | -44.9(-87.5,143.6) | 0.43 | 71.5(-15.1,246.5) | 0.14 |
| Geographical site | -52.5(-89.3,111.4) | 0.33 | 59.6(-22.2,227.5) | 0.21 |
| COVID-19 history (recovered vs. naïve) | -98.9(-99.8,-95.1) | <0.001 | -47.1(-74.3,9) | 0.09 |
| Pfizer-BioNTech | 147.6(-67.9,1810.5) | 0.39 | -0.5(-63.9,174.1) | 0.99 |
| PFHpS | -8.3(-58.9,42.3) | 0.75 | -12.1(-37.8,13.5) | 0.36 |
| Age | 0.6(-4,5.5) | 0.80 | -0.9(-3,1.3) | 0.41 |
| Female vs. male | -43.6(-87.6,156.4) | 0.46 | 60(-21.8,227.4) | 0.20 |
| Geographical site | -55.1(-90.7,115.9) | 0.32 | 45.3(-30.8,205) | 0.33 |
| COVID-19 history (recovered vs. naïve) | -98.9(-99.8,-95) | <0.001 | -45.9(-73.7,11.2) | 0.1 |
| Pfizer-BioNTech | 110.8(-73.7,1592) | 0.49 | 6(-61.7,193.6) | 0.91 |
| PFNA | -35.2(-130.8,60.3) | 0.47 | -0.8(-44.4,42.7) | 0.97 |
| Age | 0.8(-3.9,5.6) | 0.75 | -1(-3.1,1.1) | 0.34 |
| Female vs. male | -44.9(-87.7,147.9) | 0.44 | 71.3(-15.6,247.5) | 0.14 |
| Geographical site | -51.5(-89.2,118.1) | 0.35 | 59.6(-22.3,228.1) | 0.21 |
| COVID-19 history (recovered vs. naïve) | -99(-99.8,-95.3) | <0.001 | -47.1(-74.3,9.1) | 0.09 |
| Pfizer-BioNTech | 140.2(-70.7,1865.5) | 0.42 | -0.4(-64,175.8) | 0.99 |

| PFPeS | 12.4(-40.2,65.1) | 0.65 | -12(-38.5,14.5) | 0.38 |
| --- | --- | --- | --- | --- |
| Age | 0.5(-4.1,5.2) | 0.85 | -1(-3.1,1.1) | 0.34 |
| Female vs. male | -41.4(-86.9,161.8) | 0.49 | 71.9(-14.7,246.4) | 0.13 |
| Geographical site | -52.5(-89.5,114.2) | 0.34 | 61.3(-21.1,230) | 0.19 |
| COVID-19 history (recovered vs. naïve) | -98.9(-99.8,-95) | <0.001 | -48.1(-74.8,6.7) | 0.08 |
| Pfizer-BioNTech | 93.9(-74.6,1378.5) | 0.53 | 2.5(-62.7,182.2) | 0.96 |
| PFUnA | -14.4(-92.2,63.4) | 0.72 | -1.8(-39,35.4) | 0.92 |
| Age | 0.6(-4,5.4) | 0.81 | -1(-3.1,1.1) | 0.34 |
| Female vs. male | -45.3(-88.3,155.3) | 0.45 | 70.6(-16.3,247.8) | 0.14 |
| Geographical site | -50.8(-89.1,122.6) | 0.36 | 60.4(-22.6,232.7) | 0.21 |
| COVID-19 history (recovered vs. naïve) | -99(-99.8,-95.1) | <0.001 | -47.2(-74.4,9) | 0.09 |
| Pfizer-BioNTech | 108.1(-73.5,1531.7) | 0.49 | 0(-63.9,177.2) | 0.99 |
| PFECHS | -63(-152.8,26.9) | 0.17 | 3.8(-37.9,45.5) | 0.86 |
| Age | 1.2(-3.4,6.1) | 0.61 | -1.1(-3.2,1.1) | 0.33 |
| Female vs. male | -55.6(-90.4,105.9) | 0.30 | 74.5(-15.7,261.3) | 0.14 |
| Geographical site | -42.3(-87.3,161.5) | 0.48 | 57.1(-24.8,228) | 0.23 |
| COVID-19 history (recovered vs. naïve) | -99.1(-99.8,-95.6) | <0.001 | -47.1(-74.3,9) | 0.09 |
| Pfizer-BioNTech | 185(-64.4,2183.9) | 0.33 | -1.9(-64.8,173) | 0.97 |
| PFHxS | 8.9(-43.6,61.3) | 0.74 | -16.7(-43,9.5) | 0.21 |
| Age | 0.3(-4.3,5.2) | 0.89 | -0.9(-3,1.2) | 0.41 |
| Female vs. male | -38.4(-86.7,184.1) | 0.54 | 53.8(-25.1,215.8) | 0.24 |
| Geographical site | -50.5(-89.1,124.5) | 0.36 | 55(-24.1,216.6) | 0.23 |
| COVID-19 history (recovered vs. naïve) | -98.9(-99.8,-94.9) | <0.001 | -46.8(-74,9) | 0.09 |
| Pfizer-BioNTech | 81.3(-77.4,1353.7) | 0.58 | 11.7(-59.9,210.7) | 0.83 |

*Table notes.* AUC = area under the curve. CI = confidence interval. PFOA = Perfluorooctanoic acid (top). PFOS = Perfluorooctanesulfonic acid (bottom).

**Supplemental Table S6.** Weighted Quantile Sum (WQS) regression results for the change in log_2_ anti-S antibody AUC between visit 3 and baseline and between visit 4 and visit 3.

|  | Change in log_2_ anti-S antibody AUC between visit 3 and Baseline | | | | Change in log_2_ anti-S antibody AUC between visit 4 and visit 3 | | | | |  |
| --- | --- | --- | --- | --- | --- | --- | --- | --- | --- | --- |
|  | Percentage Change (95% CI) | | P-value | | | Percentage Change (95% CI) | | P-value | |  |
| Weighted Quantile Sum Score | | 24(6.5,-22) | | 0.54 | | | -11.7(6.5,-22) | | 0.25 | |
| Age | | -6.7(-12.3,0) | | 0.05 | | | -1.4(-4.1,0.7) | | 0.24 | |
| Sex | | 52.6(-84.2,1372.3) | | 0.72 | | | 89.2(-17.1,331.7) | | 0.14 | |
| Geographical site | | -82.8(-98.2,63.6) | | 0.13 | | | -1.4(-58,129.7) | | 0.97 | |
| Prior COVID-19 infection | | -88.1(-98.9,26.6) | | 0.08 | | | -45.3(-76.7,28.3) | | 0.17 | |
| Pfizer-BioNTech | | 260.5(-79.8,6344.5) | | 0.39 | | | -20.4(-73.6,139.5) | | 0.69 | |

*Table notes.* AUC = area under the curve. CI = confidence interval.

**Supplemental Figure S1.** Correlation matrix between each PFAS measured in the study population.


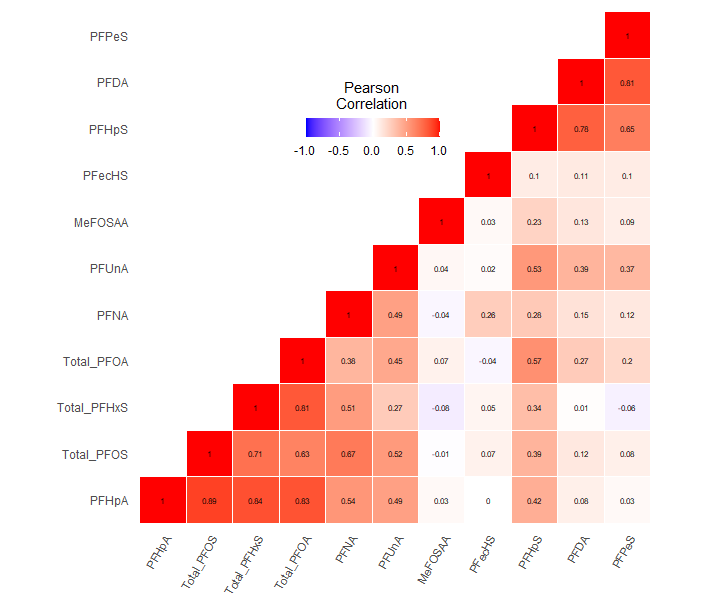


**Figure Caption.** Correlation matrix between individual log2-transformed PFAS concentrations. Interior of each box presents Pearson’s rank correlation coefficient where the darkest red indicates +1.0 and the darkest blue indicates -1.0.

**Supplemental Figure S2.** Flow chart for final sample included in analyses.

Enrolled Sample Size N=251

Removed 8 individuals due to:

- Second vaccine date unknown (either missing or incorrect, e.g., listed as the same date as the first vaccine dose).
- Second vaccine given outside of CDC’s dosing schedule (i.e., doses given more than +/- 4 days from target).

N=243ize N=251

Removed 17 individuals due to:

- Receipt of non-mRNA vaccine type (i.e., Johnson and Johnson)

Final Sample Size N=226ize N=251

**Figure caption.** Flow chart describing exclusion criteria applied to dataset, resulting in final *n* used for analyses.

**Supplemental Figure S3.** Log2 anti-s antibody AUC by day from first vaccine for PFOA and PFOS quantiles at two follow-up visits (visit 3 and visit 4).


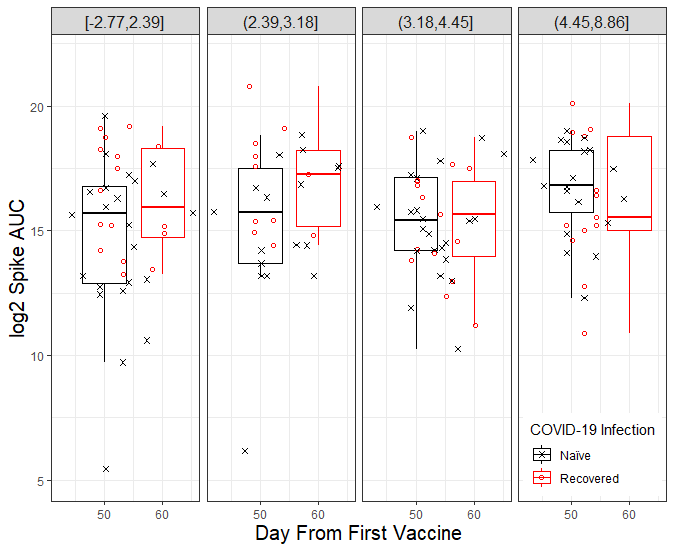

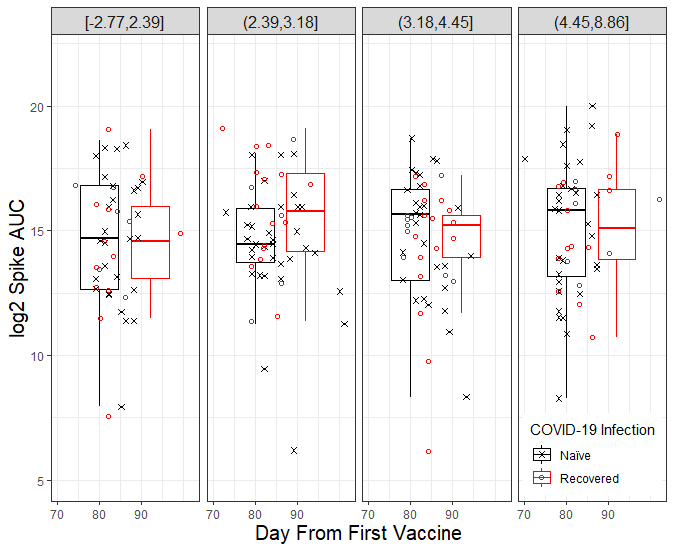

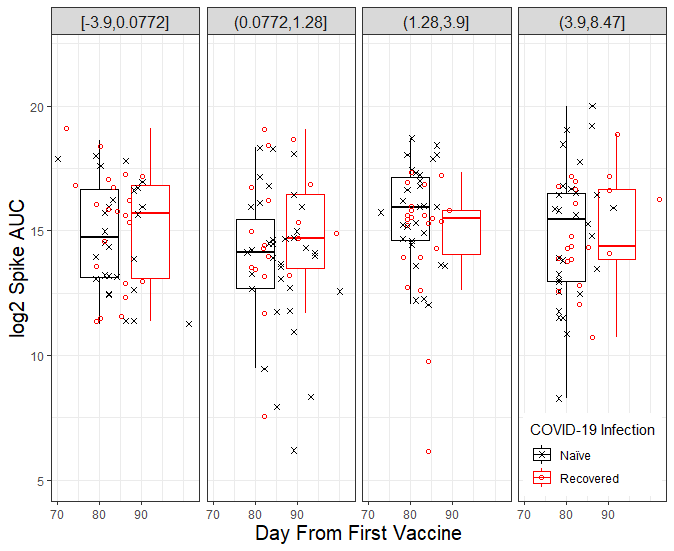

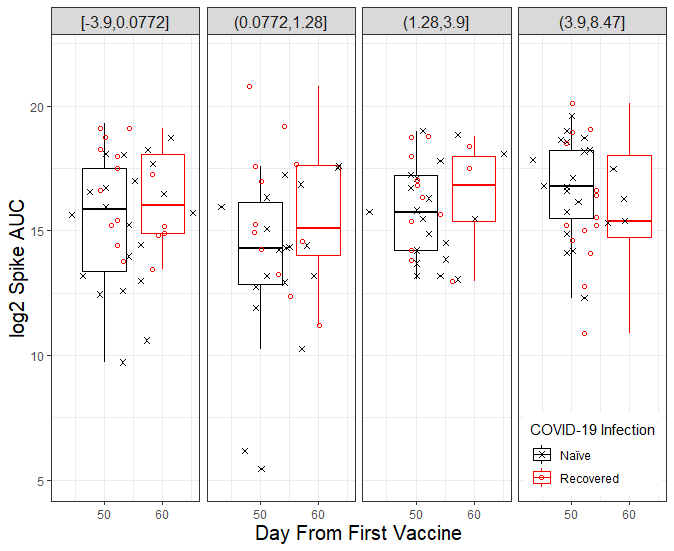


**A**

**B**

PFOA Quantiles (Visit 4)

PFOA Quantiles (Visit 3)

PFOS Quantiles (Visit 3)

PFOS Quantiles (Visit 4)

**C**

**D**

**Figure Caption.** Panels A and C correspond to data from the third follow-up visit window spanning 42 to 68 days after first vaccine dose. Panels B and D correspond to data from the third follow-up visit window spanning 42 to 68 days after first vaccine dose. Each dot represents an individual data point from participants naïve to (blue) or recovered from (red) a prior COVID-19 infection. Box plots are included for the naïve (blue) and recovered (red) groups.

**Supplemental Figure S4.** Log_2_ anti-S antibody AUC as a function of days since first vaccine dose (Pfizer-BioNTech or Moderna) for participants recovered from a prior COVID-19 infection or naïve to COVID-19. All PFAS detected among at least 60% of particpiants

log_2_ **MeFOSAA Quantiles**


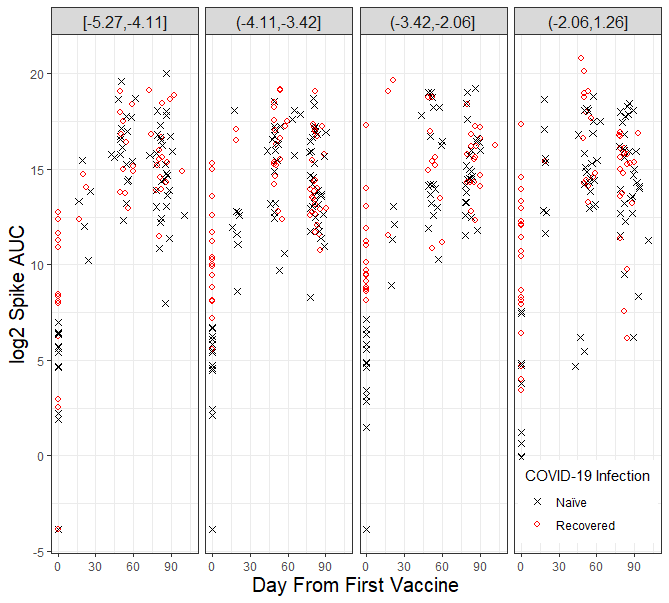


log_2_ **PFDA Quantiles**


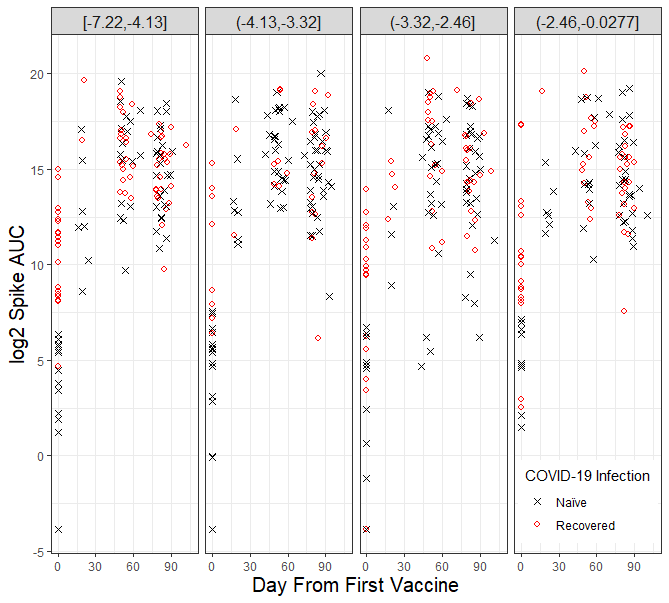


log_2_ **PFHpA Quantiles^1^**


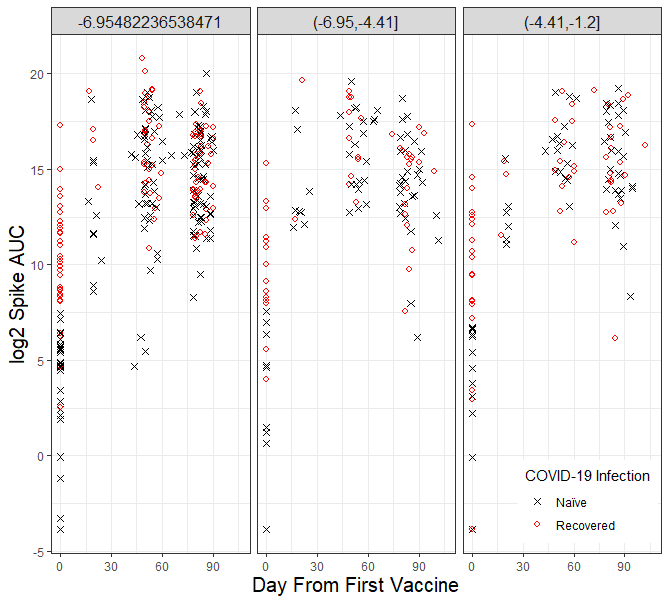


log_2_ **PFHpS Quantiles**


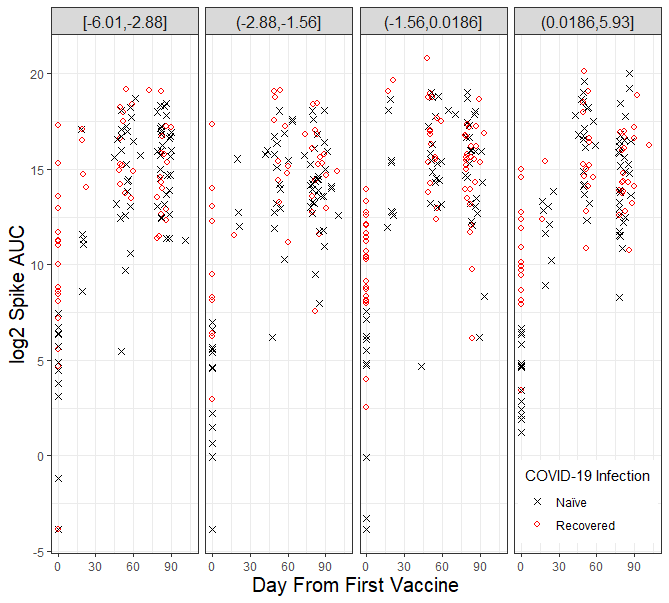


log_2_ **PFNA Quantiles**


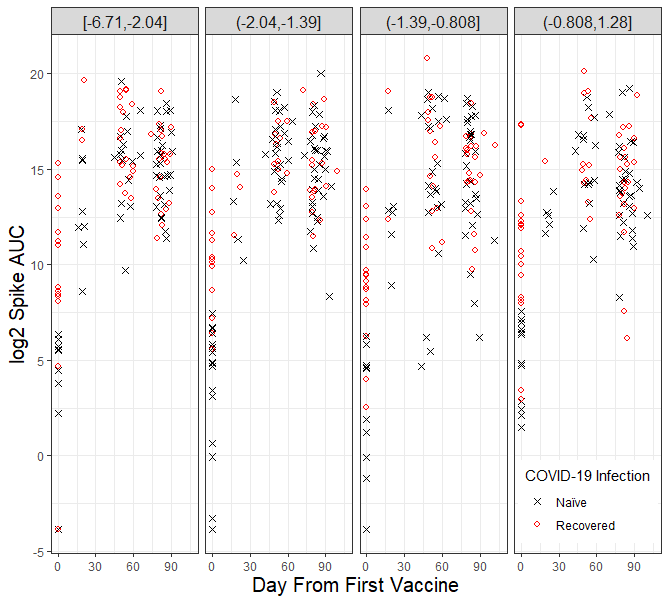


log_2_ **PFPeS Quantiles**


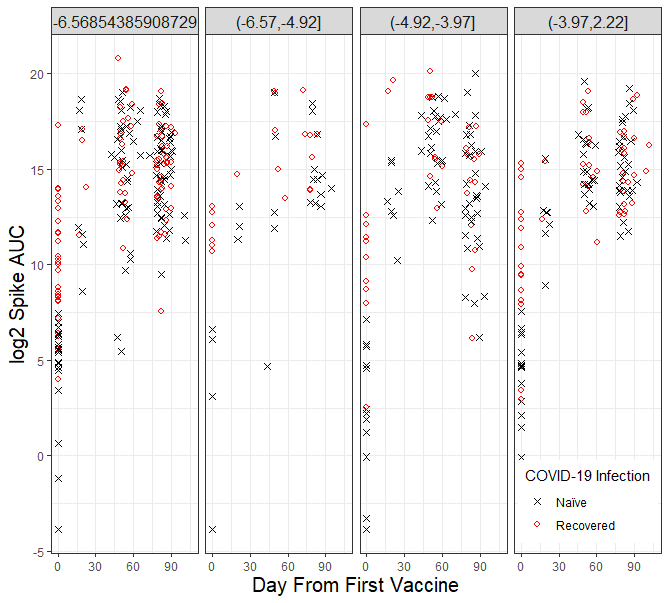


log_2_ **PFUnA Quantiles**


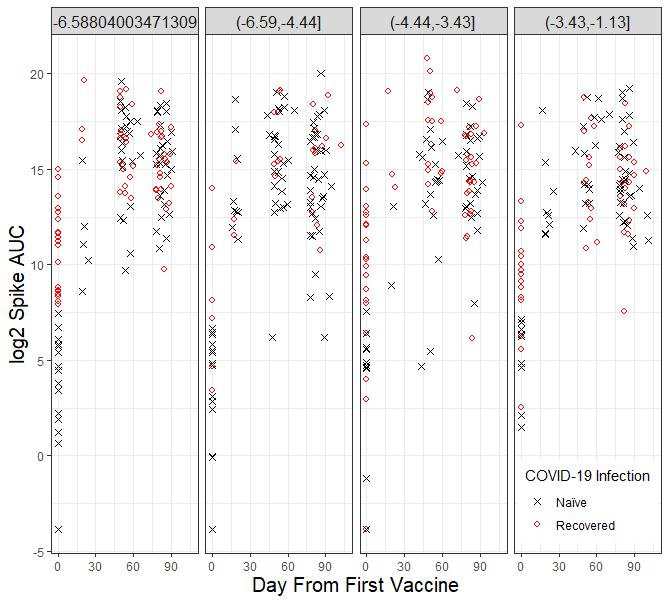


log_2_ **PFECHS Quantiles**


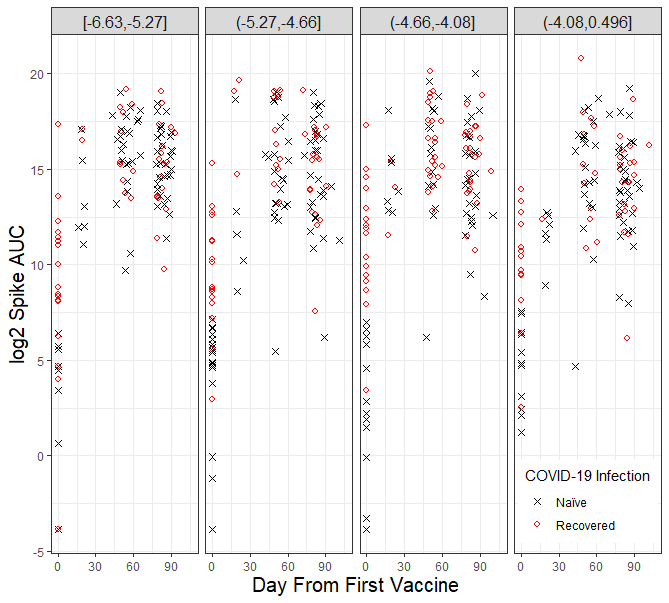


log_2_ **PFHxS (sum of L- and Br-) Quantiles**


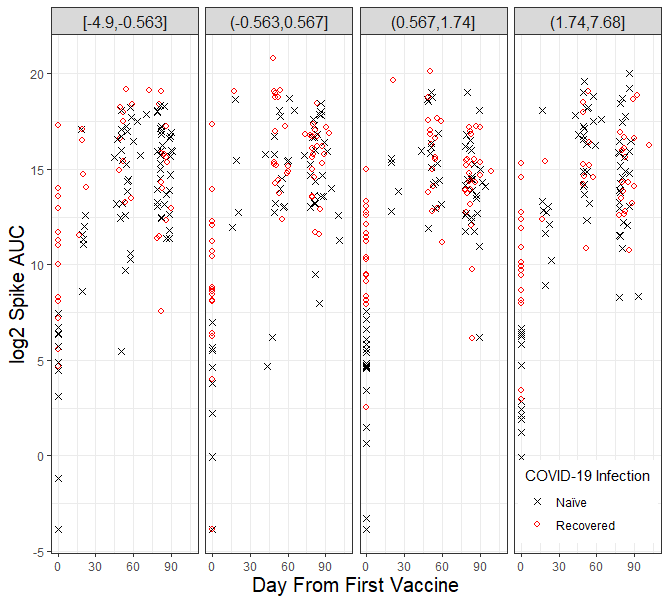


**Figure caption:** Log_2_ anti-s AUC is plotted by day from first vaccine for each quantile of PFAS for both participants naïve (blue) to prior COVID-19 infection and those recovered (red) from prior COVID-19 infection. PFAS are included if they were detected in at least 60% of participants. ^1^There are 114 (50.4%) individuals had PFHpA level under detection rate, so there are only three quantiles PFHpA.

**Supplemental Figure S5**. Relationship between PFAS and change in log_2_ anti-S antibody AUC between baseline and visit 3 and baseline and visit 4.


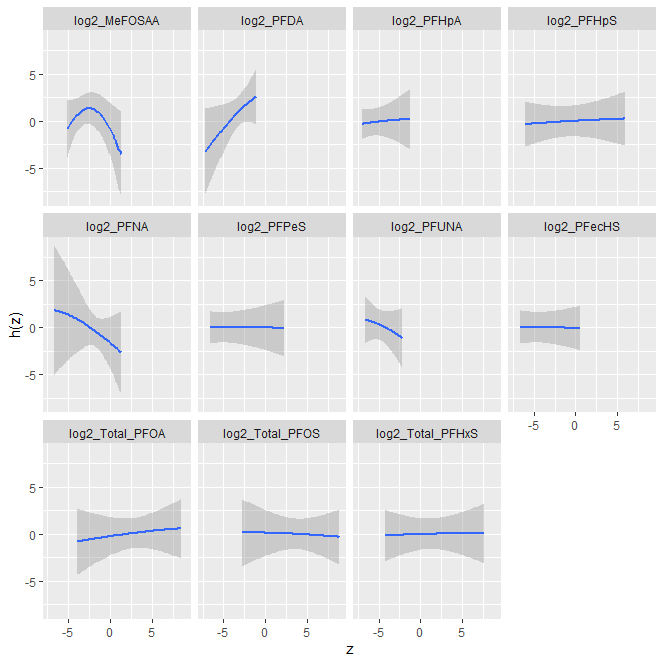

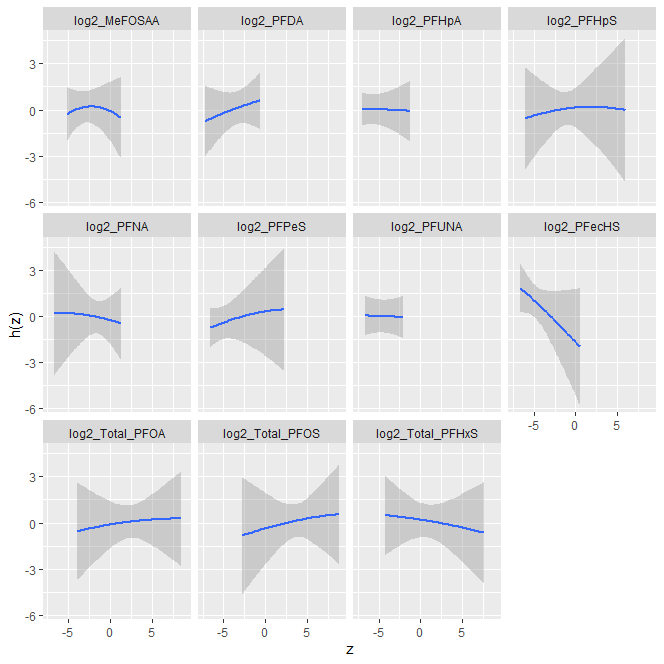


**B**

**A**

**Figure Caption**. Plots show the relationship between log_2_ PFAS chemicals with change of log_2_ anti-S antibody AUC between baseline and visit 3 (panel A) and visit 3 and visit 4 (panel B), which are all linear except MeFOSAA.
